# Population exposure across central India to PM_2.5_ derived using remotely sensed products in a three-stage statistical model

**DOI:** 10.1038/s41598-020-79229-7

**Published:** 2021-01-12

**Authors:** Prem Maheshwarkar, Ramya Sunder Raman

**Affiliations:** 1 Department of Earth and Environmental Sciences, Indian Institute of Science Education and Research Bhopal, Bhopal Bypass Road, Bhauri, Bhopal, Madhya Pradesh 462 066 India; 2 Center for Research on Environment and Sustainable Technologies, Indian Institute of Science Education and Research Bhopal, Bhopal Bypass Road, Bhauri, Bhopal, Madhya Pradesh 462 066 India

**Keywords:** Atmospheric chemistry, Atmospheric dynamics

## Abstract

Surface PM_2.5_ concentrations are required for exposure assessment studies. Remotely sensed Aerosol Optical Depth (AOD) has been used to derive PM_2.5_ where ground data is unavailable. However, two key challenges in estimating surface PM_2.5_ from AOD using statistical models are (i) Satellite data gaps, and (ii) spatio-temporal variability in AOD-PM_2.5_ relationships. In this study, we estimated spatially continuous (0.03° × 0.03°) daily surface PM_2.5_ concentrations using MAIAC AOD over Madhya Pradesh (MP), central India for 2018 and 2019, and validated our results against surface measurements. Daily MAIAC AOD gaps were filled using MERRA-2 AOD. Imputed AOD together with MERRA-2 meteorology and land use information were then used to develop a linear mixed effect (LME) model. Finally, a geographically weighted regression was developed using the LME output to capture spatial variability in AOD-PM_2.5_ relationship. Final Cross-Validation (CV) correlation coefficient, r^2^, between modelled and observed PM_2.5_ varied from 0.359 to 0.689 while the Root Mean Squared Error (RMSE) varied from 15.83 to 35.85 µg m^−3^, over the entire study region during the study period. Strong seasonality was observed with winter seasons (2018 and 2019) PM_2.5_ concentration (mean value 82.54 µg m^−3^) being the highest and monsoon seasons being the lowest (mean value of 32.10 µg m^−3^). Our results show that MP had a mean PM_2.5_ concentration of 58.19 µg m^−3^ and 56.32 µg m^−3^ for 2018 and 2019, respectively, which likely caused total premature deaths of 0.106 million (0.086, 0.128) at the 95% confidence interval including 0.056 million (0.045, 0.067) deaths due to Ischemic Heart Disease (IHD), 0.037 million (0.031, 0.045) due to strokes, 0.012 million (0.009, 0.014) due to Chronic Obstructive Pulmonary Disease (COPD), and 1.2 thousand (1.0, 1.5) due to lung cancer (LNC) during this period.

## Introduction

Increased cardiovascular and respiratory diseases in addition to a decreased life expectancy are associated with chronic exposure to particulate matter with aerodynamic diameters < 2.5 µm, PM_2.5_^1^. The Global Burden of Disease (GBD) 2015 study identified air pollution as a major cause of global disease burden, with low and middle-income countries being the worst affected^2^. In India, PM_2.5_ standards were included in the National Ambient Air Quality Standards (NAAQS) in November 2009 and are monitored by the central and several state pollution control boards. However, PM_2.5_ concentrations vary in space over sub-kilometer to continental scales^3^. The current surface PM_2.5_ monitoring network in India is inadequate to capture this variability and to provide adequate data for population exposure studies. Satellite retrieval proxies of PM_2.5_ such as Aerosol Optical Depth (AOD), which is the measure of the overall light extinction attributed to the aerosols in the atmospheric column, has been extensively used to estimate surface fine aerosols concentrations worldwide^4–7^. During the mid-2000s, various studies estimated surface PM_2.5_ concentrations by establishing a linear relationship between AOD and surface PM_2.5_. Another popular approach was by using η factor (the ratio of PM_2.5_ modelled and AOD modelled) obtained from various global Chemical Transport Models (CTMs) such as GEOS Chem as a scaling factor to convert satellite-derived AOD to surface PM_2.5_ concentration^8,9^. During the last decade or so, several studies estimated the global concentrations of surface PM_2.5_ using satellite-derived AOD and η factor obtained from various CTMs^10,11^ as this method does not require surface measurements to develop the model. However, results from these studies for locations in India were not properly validated, due to the lack of monitoring stations in India at the time that these studies were conducted.

Recently, a number of statistical models were developed to capture the varying relationships between AOD and surface PM_2.5_ concentrations at various locations across the world, such as the linear mixed effect model, geographically weighted regression, and generalized additive models^12,13^. These studies have used meteorological parameters (height of planetary boundary layer, surface temperature, wind speed, relative humidity) and land use information as covariates along with satellite AOD to estimate surface PM_2.5_ concentrations. Results from these studies have shown that meteorological fields and land use information improve the model performance significantly. More sophisticated models were then developed by combining two or more regression models to hierarchically estimate the surface PM_2.5_ concentrations^14,15^. These models were usually generated by combining linear mixed-effects models in the first stage with generalized additive models or geographically weighted regression models in the second stage to capture the spatiotemporal variation in the relationship between AOD, PM_2.5_, and meteorological parameters. These hybrid models have shown a strong correlation between estimated and measured PM_2.5_ mass concentration worldwide with improved performance when compared to individual models. Another major challenge in estimating spatially continuous PM_2.5_ arises due to spatially non-continuous AOD values owing to cloud coverage, rainfall, and satellite calibrations. Previous studies in the literature have tried to fill MODIS AOD data by using random forest algorithm^16,17^, spatiotemporal regression kriging^18^ and by using two-staged generalized additive model^19^. However, these studies have their own limitations and specific pre-requisites, limiting model application to real-life situations.

In India, studies to estimate surface PM_2.5_ concentration using satellite proxies are still in an embryonic stage with very few national studies^13,20–23^ reporting low r^2^ values (Supplemental Table S1) between measured and estimated PM_2.5_. Due to the lack of extensive ground PM_2.5_ measurements in India, very few studies use empirical statistical models, of which a majority were developed for the Delhi region^24,25^. A recent study^23^ estimated PM_2.5_ concentration over India for January–August 2017 using spatiotemporal mixed effect models, and chose ordinary spatial Kriging, inverse distance weighting (IDW) and spline interpolation to estimate PM_2.5_ concentration over grids with no AOD values and reported that spline interpolation performed better than IDW and Kriging. Further, none of these studies validated their model performances against surface PM_2.5_ data over Madhya Pradesh (MP) or other states in central India. Current estimates of spatially continuous PM_2.5_ concentration over MP are derived from global studies such as (van Donkelaar et al.^11^ and van Donkelaar et al.^10^), for various epidemiological and GBD studies. These estimates from CTMs in conjunction with satellite-derived AOD are strongly influenced by model chemistry, physical processes, and emissions inventory, all of which may in-turn fail to capture the ground realities^26^. CTMs in general, do not incorporate all of the complexities in aerosol mixing states (which are only beginning to be understood) and thus the (η) factor approach often provides biased surface PM_2.5_ estimates.

The goal of this study is to develop a three-stage statistical model to capture spatio-temporal variability in AOD-PM_2.5_ relationship, in order to estimate spatially continuous surface PM_2.5_ concentrations over MP state, central India, for 2018 and 2019. This endeavor is made possible by the recently available Central Pollution Control Board (CPCB) India surface PM_2.5_ over several locations in the state. We take a three-step approach to achieve our study goals. In the first step, missing Multi-Angle Implementation of Atmospheric Correction (MAIAC) AOD values were imputed, using yearly grid-wise linear regression between MAIAC AOD and the Modern-Era Retrospective analysis for Research and Applications (MERRA-2) derived AOD. Imputed AOD and MEERA-2 meteorological parameters in conjunction with land use variables were then used to develop a linear mixed effect model (LME) to capture the daily variability in the relationship between AOD, PM_2.5_ and meteorological parameters. Finally, to capture the spatial variability in AOD-PM_2.5_ relationship a Geographically Weighted Regression model (GWR) was developed. The annual PM_2.5_ concentrations thus obtained were then compared against ground measurements and PM_2.5_ concentrations obtained from a recent study incorporating advances in CTM implementation and using a GWR (Hammer et al.^27^). An additional objective of this study was to utilize the derived concentrations to estimate the population exposure to PM_2.5_ and the associated premature mortality over Madhya Pradesh during 2018–2019.

## Study area

**Figure 1:**
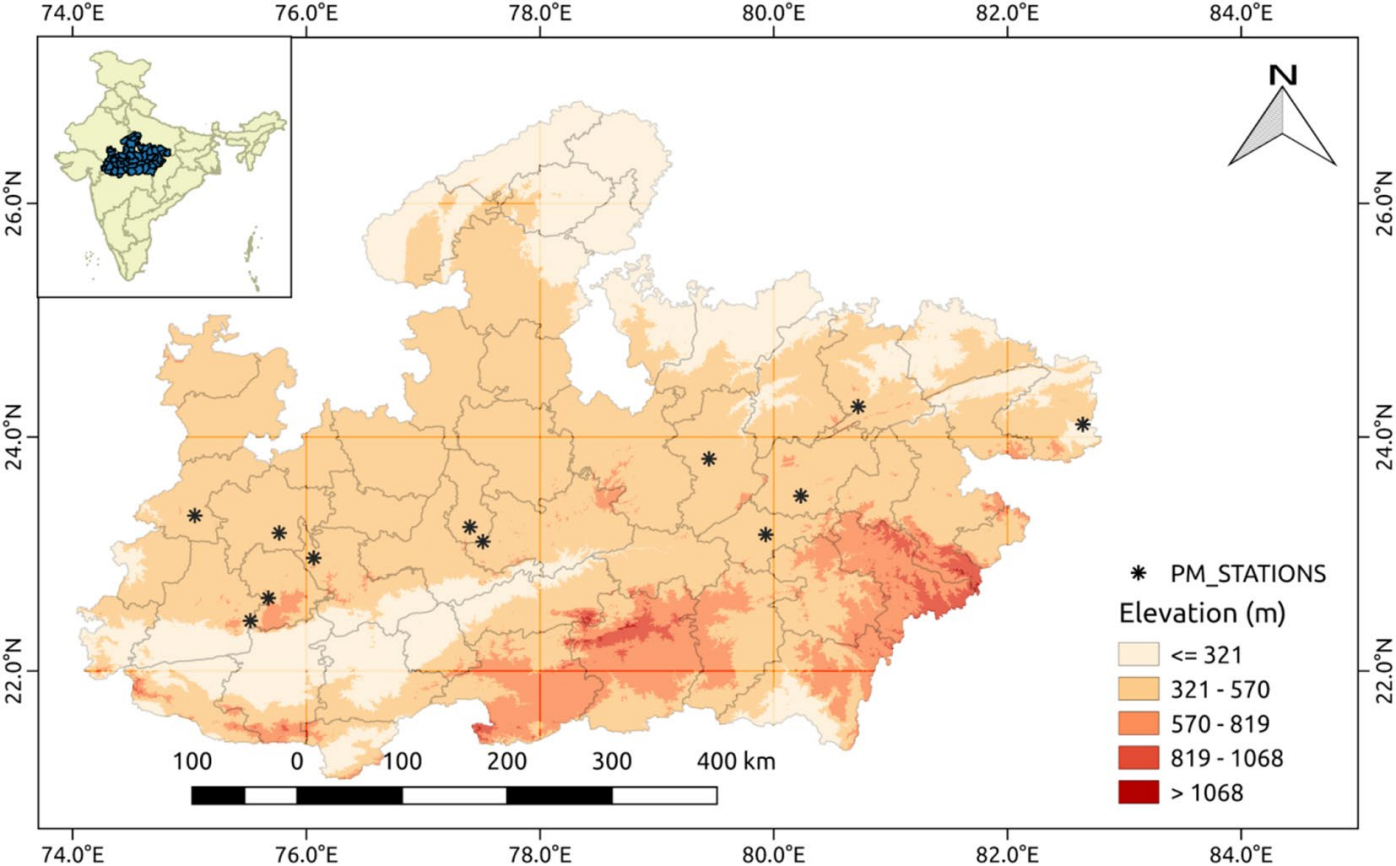
Elevation map (amsl) of the study area and location of PM_2.5_ monitors in MP state. This map is generated using QGIS 2.18.1 (http://www.qgis.org).

This study was conducted over MP state (Fig. 1) in central India [27 N, 74E–21 N, 84E]. MP is the second largest state in India by area with a total geographical area of 3,08,245 km^2^ and the fifth-largest state by population Census 2011^28^. The northeastern boundary of MP is lined by Indo Gangetic Plain (IGP), one of the most air-polluted regions in the world^10,11^. Previous studies have shown 24 h mean PM_2.5_ mass concentrations of up to 170 μg m^−3^ in the IGP and in parts of MP^23^. Based on the Indian Meteorological Department classification, MP has four distinct seasons: winter (Jan, Feb), pre-monsoon (Mar, Apr, May), monsoon (Jun, Jul, Aug, Sep) and post-monsoon (Oct, Nov, Dec) and has a mixture of semi-arid, tropical, and subtropical climate. The annual mean temperature over MP is 24.7 °C with an average daily high temperature of 33 °C (averages over the last 20 years). MP receives most of its rainfall in the monsoon season with a mean rainfall of 1160 mm with high spatial variability (rainfall decreases from east to west). The mean elevation of MP ranges between 72 m amsl and 1317 m amsl.

## Material and methods

### Ground PM_2.5_ measurement

The Central Pollution Control Board (CPCB) monitors ambient air quality under a nation-wide program: National Ambient Air Quality Monitoring Programme (NAMP). The monitoring stations report PM_2.5_ mass concentration in μg m^−3^ at 15 min resolution, measured using the tapered element oscillating microbalance (TEOM) or the beta-attenuation method (BAM) (CPCB 2011)^29^. Daily mean PM_2.5_ concentration data were thus obtained for 12 stations in different cities across MP for the period between January 2018 and December 2019 from the CPCB database (https://app.cpcbccr.com/ccr/#/caaqm-dashboard-all/caaqm-landing). The location of air monitoring stations are shown in Fig. 1 and data completeness over these stations is shown in Supplemental Figure S1.

### MODIS AOD

The MODerate resolution Imaging Spectroradiometer (MODIS) sensors onboard Earth Observation System (EOS) Terra and Aqua were launched to Sun-synchronous polar orbits in late 1999 and in 2002, having satellite overpass times over India at 10:30 a.m. and 01:30 p.m., respectively. They have a circular orbit of 705 km and a swath of approximately 2330 km. Daily measurements across a wide spectral range yields multiple datasets of AOD and a variety of other products. In this study, we have used the Multi-Angle Implementation of Atmospheric Correction (MAIAC) for a combined data product of Terra and Aqua AOD. MAIAC algorithm was developed for MODIS data to perform the retrieval of aerosols and for atmospheric correction over bright and dark surfaces (vegetated) utilizing image processing and time-series analysis to derive bidirectional reflectance distribution function at a resolution of 1 km^30^. Due to clear surface characterization, MAIAC AOD has lesser urban bias and increased spatial coverage when compared to the dark target AOD products^31^. Previous studies in the literature have successfully estimated daily PM_2.5_ concentrations by utilizing Terra and Aqua AOD averages^14,32^. MAIAC files contain multiple (2–4) AOD files per day depending upon the number of Terra and Aqua overpasses. Due to changing cloud cover during a day, there is a difference in the spatial and temporal availability in AOD data per day. Availability of MAIAC AOD over the location of surface stations in MP is given in Supplemental Figure S2.

### MERRA-2 AOD

The Modern-Era Retrospective analysis for Research and Applications version 2 (MERRA-2) is a NASA atmospheric reanalysis using the Goddard Earth Observing System Model, Version 5 (GEOS-5) coupled with GOCART aerosol module with its Atmospheric Data Assimilation System (ADAS), version 5.12.4. MERRA-2 reanalysis data is available from 1980-present, globally. MERRA-2 aerosol analysis uses the GEOS-5 Aerosol Assimilation System (GAAS) and assimilates bias-corrected AOD from the ground and satellite-based instruments such as MODIS, MISR, AVHRR and AERONET^33^. MEERA-2 AOD has been validated against MODIS, MISR AOD and in situ AOD worldwide and has shown good agreement with measured AOD^33,34^. In India, daily mean MERRA-2 AOD at 550 nm compared well (r = 0.79) with AERONET AOD measured over Kanpur during 2011–2016^35^. In this study, we obtained hourly total aerosol extinction at 550 nm from MERRA 2 aerosol diagnostics at a resolution of 0.5° × 0.625° (latitude × longitude) over MP state for 2018 and 2019. The dataset was then extracted for 05:00 UTC to 08:00 UTC (10:30 a.m.–01:30 p.m. IST) to match with the MAIAC AOD data.

### Meteorological data

Hourly meteorological dataset: air temperature at 2 m, relative humidity as 2 m, the eastward and northward component of wind velocity as 10 m above the surface level and surface pressure were obtained from MERRA-2 single-level diagnostics and height of planetary boundary layer in meters was obtained from MERRA-2 surface flux diagnostics. These datasets were obtained hourly from 05:00 UTC to 08:00 UTC for the study period at a spatial resolution of 0.625° × 0.5° from GES-DISC over MP.

### Land-cover and road-density data

Shapefile for the major roadways map for India was obtained from https://mapcruzin.com/. This shapefile was then clipped to match the state boundaries of MP. The density of the road network in a grid cell was obtained by dividing the length of roads in a grid by the total area of the defined grid cell. Land use and land cover map for 2018 over India was downloaded from European Centre for Medium-Range Weather Forecasts (ECMWF) at a spatial resolution of 300 m × 300 m. Annual land cover classification gridded maps are available from 1992 to 2019 and have divided land cover in 22 classes defined by the United Nations Food and Agriculture Organization’s (UN FAO) Land Cover Classification System (LCCS). In-depth documentation on the algorithms used to derive the land cover can be accessed via maps.elie.ucl.ac.be/CCI/viewer/download/ESACCI-LC-Ph2-PUGv2_2.0.pdf. Out of these 22 classes available, grid cells of urban cover, cropland and grassland cover were extracted for use in this study.

### Data integration

In this study, PM_2.5_ ground measurements were point data. Additionally, four different gridded datasets were used: the MAIAC AOD obtained from LAADS DAAC in HDF format; daily mean MERRA-2 AOD and meteorological parameters obtained from GES DISC originally stored in NetCDF; and land cover classification gridded maps obtained from ECMWF. Additionally, the road map of Madhya Pradesh was in a shapefile format. A brief description of these datasets and their source is given in Table 1.

**Table 1:** List of all input parameters used in the statistical model.

Parameters	Temporal resolution	Resolution	Sensor	Type of data	Data period	Source
Surface PM_2.5_	Daily	Point data	TEOM/BAM	In-situ	2018–2019	CPCB
AOD	Daily	1 km × 1 km	MODIS	Satellite	2018–2019	LAADSDAAC
	Daily	0.625° × 0.5°	MERRA-2	Reanalysis	2018–2019	GES DISC
Meteorological parameters	Daily	0.625° × 0.5°	MERRA-2	Reanalysis	2018–2019	GES DISC
Land use land cover	Yearly	300 m × 300 m	Copernicus	Satellite	2018	ECMWF
Road map	Yearly	–		Shapefile	2018	

To maintain a reasonable file size MAIAC AOD data is stored in 1200 km × 1200 km tiled file structure. Madhya Pradesh is covered by two tiles i.e. h25v06 and h24v06. These files contain multiple AOD files per day (2–4) from multiple overpasses of Terra and Aqua platform. Daily mean AOD values from these multiple swaths were calculated for each grid and were clipped for MP state. MAIAC AOD data was available in curvilinear projection and this had to be remapped to rectilinear projection for obtaining a consistent spatiotemporal dataset with MERRA-2 AOD and meteorological parameters (originally in rectilinear projection). AOD re-mapping is time-consuming and computationally expensive, therefore, to balance between spatial resolution and computational time, the original curvilinear 1 km × 1 km MAIAC AOD was remapped to 0.03° × 0.03° rectilinear projection. Previous studies in the literature have adopted a similar remapping technique to estimate AOD at a lower resolution. Ma et al.^36,37^ remapped MODIS C6 AOD (3 km × 3 km) to 0.1° × 0.1° and MODIS C5.1 (10 km × 10 km) to 50 km resolution data over China, respectively. vanDonkelaar et al.^11^ remapped global MAIAC AOD to regional 0.1° × 0.1° and 0.01° × 0.01° to estimate the global burden of surface PM_2.5_ concentrations. Meanwhile, a bilinear-interpolation method was used for MERRA-2 AOD and meteorological parameters to match the spatial resolution with gridded MAIAC data. For stations like Singrauli, PM_2.5_ mass concentration was as high as 800–1000 μg m^−3^ for a few days (Supplemental Table S2). Very high episodic PM_2.5_ concentration days were outliers and not reflected by unusually high AOD over these stations on such days. Therefore PM_2.5_ concentration values greater than 99.9th percentile were not used in model development or validation. Gridded road density network (Supplemental Figure S3) was obtained using the method described in “Land-cover and road-density data” section. Finally, the number of grid cells of urban cover, grassland and cropland falling in the predefined 0.03° × 0.03° grid were calculated.

### Model development

#### Stage 1: imputing missing MAIAC AOD with MERRA-2 AOD

MODIS data has often missing AOD values due to cloud coverage, monsoons and satellite calibration issues. Spatial distribution of the percentage of annual mean available data days for 2018 and 2019 are shown in Supplemental Figure S4. Percentage of daily MAIAC AOD available over MP ranged from 0.0 to 73.15% with a mean value of 59.47% and 0.0% to 63.83% with a mean value of 50.56% during 2018 and 2019, respectively. There are no specific spatial patterns in missing data, except almost no data available over large water bodies in MP. To fill daily MAIAC AOD data gaps, first, grid-wise linear regression was fitted between daily pixel centroid values of re-sampled 0.03° MAIAC AOD and 0.03° MERRA-2 AOD for each year to obtain regression coefficients for every grid (201 × 334 = 67,134 in total). The missing MAIAC AOD_(s,t)_ on day “t” and grid point “s” was then filled using the regression coefficient obtained for grid “s” from the first step and MERRA-2 AOD_(s,t)_ over that grid point on day “t” as shown in Eq. (1).

1 $${\text{Final}}\_{\text{AOD}}_{{{\text{s}},{\text{t}}}} = \upalpha_{{\text{s}}} + \upbeta_{{\text{s}}} \, \times {\text{ MERRA2}}\_{\text{AOD}}_{{{\text{s}},{\text{t}}}}$$

where Final_AOD_s,t_ is the imputed AOD over grid s on day t provided MAIAC_s,t_ is unavailable; α_s_ and β_s_ are the coefficient obtained from linear regression between daily MAIAC AOD and MERRA-2 AOD over grid s; and MERRA2_AOD_s,t_ is the MERRA-2 AOD over grid s on day t.

MERRA-2 AOD was able to capture the temporal variation in MAIAC AOD with a mean temporal r^2^ value of 0.63 and 0.61 during 2018 and 2019, respectively over the MP region. Grid-wise temporal r^2^ between MAIAC and MERRA-2 AODs and range of r^2^ values are provided in Supplemental Figures S5 and S6. However, MERRA-2 consistently under estimated AOD value throughout the study period compared to MAIAC AOD (slope and intercept of the regression equations are provided in Supplemental Figures S7 and S8). We also fitted seasonal linear regression between daily MAIAC and MERRA-2 AOD to estimate seasonal slope and intercept along with the corresponding p-values (Supplemental Text S1 and Supplemental Figures S9–S12). For the monsoon season, the p-values for the slope were greater than 0.01 for a substantial number of grid points during both 2018 and 2019 (Supplemental Figures S9 and S10) bringing down the confidence in imputed AOD. Therefore in the final imputation, estimates of slope and intercept from yearly regression, as shown in Eq. (1) were used.

#### Stage 2: linear mixed effect model

In the second stage, a linear mixed-effect (LME) model was developed following the approach proposed by Lee et al.^38^, over the New England region in the USA. A LME model captures daily variability in the relationship between AOD, PM_2.5,_ and meteorological parameters. Day-specific slopes and intercepts for the relationship between PM_2.5,_ AOD and meteorological parameters are calculated and incorporated into both fixed-effects terms and random-effects terms in a LME model. Several studies have shown that the relationship between AOD and surface PM_2.5_ varies with changing meteorology due to changing PM_2.5_ vertical profile, hygroscopic particle growth and optical properties^15,36^ . Also spatially changing parameters such as urban cover, forest cover and vegetation can also affect AOD-PM_2.5_ relationship due to changing sources and chemical composition of PM_2.5_^36^. Therefore, in this study, we used meteorological parameters along with land cover variables as independent variables and surface PM_2.5_ as a dependent variable to develop a LME model in the second stage, which can be written as:

2 $$\begin{aligned} {\text{PM}}_{{{2}.{5}{\text{ (s,t)}}}} & = \left( {{\text{a}}_{0} + {\text{a}}_{{0,{\text{t}}}} } \right) \, + \, \left( {{\text{a}}_{{1}} + {\text{ a}}_{{{1},{\text{t}}}} } \right){\text{AOD}}_{{{\text{s}},{\text{t}}}} + \, \left( {{\text{a}}_{{2}} + {\text{ a}}_{{{2},{\text{t}}}} } \right){\text{Temp}}_{{{\text{s}},{\text{t}}}} \hfill \\ & \quad + \, \left( {{\text{a}}_{{3}} + {\text{a}}_{{{3},{\text{t}}}} } \right){\text{RH}}_{{{\text{s}},{\text{t}}}} + \left( {{\text{a}}_{{4}} + {\text{a}}_{{{4},{\text{t}}}} } \right){\text{U1}}0_{{{\text{s}},{\text{t}}}} + \, \left( {{\text{a}}_{{5}} + {\text{a}}_{{{5},{\text{t}}}} } \right){\text{ Pressure}}_{{{\text{s}},{\text{t}}}} \hfill \\ & \quad + {\text{ a}}_{{6}} {\text{UrbanCovers }} + {\text{ a}}_{{7}} {\text{GrassLand }} + \, \varepsilon_{{{\text{st}}}} \left( {{\text{a}}_{{0,{\text{t}}}} ,{\text{ a}}_{{{1},{\text{t}}}} ,{\text{ a}}_{{{2},{\text{t}}}} ,{\text{ a}}_{{{3},{\text{t}}}} ,{\text{ a}}_{{{4},{\text{t}}}} ,{\text{a}}_{{{5},{\text{t}}}} } \right) \, \sim {\text{N}}\left[ {\left( {0,0,0,0,0,0} \right),\Sigma } \right] \hfill \\ \end{aligned}$$

where PM_2.5(s,t)_ is the surface PM_2.5_ concentration in (µg m^−3^) over grid s on day t: a_0_ and a_0,t_ are fixed and random (daily varying) intercept, respectively: AOD_s,t_ is the imputed AOD (unitless) over grid s on day t. RH_s,t_, Temp_s,t_, U10_s,t_, Pressure_s,t_ are relative humidity and temperature (℃) at 2 m above ground level, U10 is the eastward component of wind speed at 10 m above the ground level and Pressure is the surface pressure over grid s on day t. UrbanCover and GrassLand are the number of grid points of urban cover and grass-land available inside the grid s. (a_1_–a_7_) represents the fixed slopes of variables over the entire study period while (a_1,t_–a_5,t_) are the changing slopes with day t. ε_st_ is the error term on grid s on day t and Σ is the variance–covariance matrix for the random effects. Additional predictors such as road density, the daily height of planetary boundary layer, crop-land cover were also included in the LME model development. But the slope estimate values were not statistically significant therefore they were not included in the final model given by Eq. (2).

#### Stage 3: geographically weighted regression

In the final stage, a Geographically Weighted Regression (GWR) model was developed to capture the spatially varying relationships between AOD and PM_2.5_ using the output from the LME model. A GWR model captures spatial heterogeneity by generating a continuous surface of model parameters at every grid cell instead of universal value for all observations (predictor and response variable). We fitted a daily Gaussian GWR model using adaptive bandwidth selection to minimize the Akaike Information Criterion (AIC_c_) value using adaptive bi-square kernel in MGWR python^39^ given by Eq. (3).

3 $${\text{PM2}}.{5}\_{\text{residual}}_{{{\text{s}},{\text{t}}}} = {\text{ b}}_{{0,{\text{s}}}} + {\text{ b}}_{{{1},{\text{s}}}} {\text{AODs}},{\text{t }} + \varepsilon^{{1}}{_{{{\text{st}}}}}$$

where PM2.5_residual_s,t_ is the residual PM_2.5_ concentration (observed–estimated) obtained after fitting the LME model over grid s and day t, AOD_s,t_ is the imputed AOD (unitless) over grid s and day t: b_0,s_ and b_1,s_ are location specific intercept and slope over grid s, respectively, which is a function of the geographic location, and ε^1^_st_ is the error term over grid s and day t.

To assess the model fit performance, statistical indicators (r^2^, Root Mean Square Error (RMSE) and Mean Absolute Error (MAE)) were used to estimate the goodness of fit for both stage-2 and stage-3 models. Furthermore, to avoid any model overfitting, a tenfold Cross-Validation (CV) approach was used after stage-2 and stage-3 to estimate the overall model performance. In a tenfold CV, the entire dataset of 4922 observations was divided into 10 random sub-groups (~ 492 points each), and data from 9 sub-groups were used to train the model. The remaining group was used for model validation. This validation scheme is repeated ten times until every subgroup is validated. The metrics were then calculated by comparing PM_2.5_ observations and estimates that are collected from all 10 subgroups. Furthermore, due to unavailability of AERONET stations or any campaign mode in situ AOD measurement over MP, to estimate the performance of imputed AOD using MERRA-2 AOD we chose days over study area where MAIAC AOD data was unavailable but surface PM_2.5_ data were available. We then checked final model performance on such days against surface concentration to assess the performance of imputed AOD to estimate PM_2.5_ concentration.

## Results and discussion

### Descriptive statistics

Percentage frequency distribution of AOD, PM_2.5_, and meteorological variables used in the statistical models are summarized in Supplemental Figure S13. MAIAC AOD, MERRA-2 AOD and PM_2.5_ follow a similar distribution, indicating that these three variables are indeed related to each other. The mean MAIAC AOD over surface PM_2.5_ monitoring stations for the study period was 0.4034 and corresponding MERRA-2 AOD and surface PM_2.5_ concentrations were 0.3482 and 67.89 µg m^−3^, respectively. MERRA-2 AOD consistently underestimated the AOD over Madhya Pradesh compared to MAIAC AOD. Temperature and relative humidity show significant seasonality (Supplemental Figure S13) as suggested by their bimodal distributions.

Seasons are defined following the Indian Meteorological Department (IMD) classification for this region as winter (Jan, Feb), pre-monsoon (Mar, Apr, May), monsoon (Jun, Jul, Aug, Sep) and post-monsoon (Oct, Nov, Dec). Seasonal variation in AOD over MP was not statistically significant (Supplemental Table S2). Further, the 2009 NAAQS over India were revised to include the daily and annual PM_2.5_ mass concentration standards with values of 60 μg m^−3^ and 40 μg m^−3^, respectively. PM_2.5_ mass concentrations measured over CPCB stations in MP exceeded the daily average standard more than 33.49% of the days, for which data were available, during the study period.

### Model fitting and validation

The fixed effect terms of all the variables from stage-2 after fitting the LME model are summarized in Table 2. The PM_2.5_ concentration increases with increasing AOD, surface pressure, urban cover, grassland cover and northward winds in a grid cell and decreases with increasing temperature and relative humidity. Detailed results from the model are provided in Supplemental Table S3. ECMFW LULC classifies Mosaic herbaceous cover (> 50%)/tree and shrub (< 50%) and grassland as grassland. This herbaceous cover land becomes open/dry land during summer/dry season and could potentially contribute dust aerosol to PM_2.5_ loading in MP giving a positive slope with PM_2.5_.

**Table 2:** Fixed effect terms (intercept and slope estimates) for LME model after stage-2.

Variable	Coefficient	p-value
Intercept	− 823.031	< 0.001
Imputed AOD (unitless)	34.833	< 0.001
V10 (m/s)	− 1.178	< 0.001
Temperature (℃)	− 1.104	< 0.001
RH (100)	− 23.502	< 0.001
Pressure (Pa)	0.009	< 0.001
Urban	0.100	< 0.001
Grassland	0.796	< 0.001

**Figure 2:**
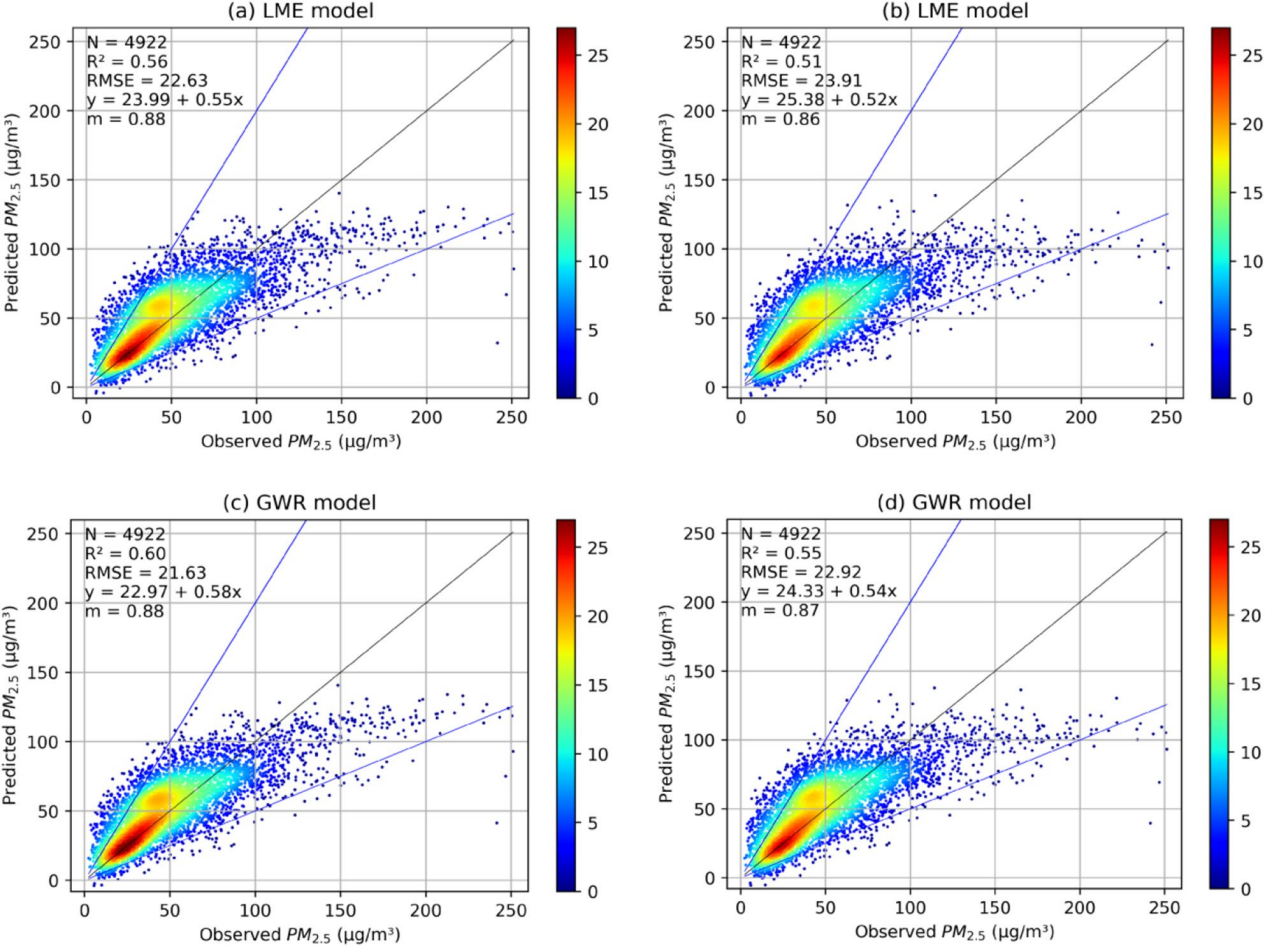
(**a**) LME and (**b**) LME are Model training and tenfold cross-validation of LME model, respectively over Madhya Pradesh during 2018–2019 and (**c**) GWR is GWR model training and (**d**) GWR is after tenfold CV (caxis is the point count). The blue lines are the y = 2 × and y = x/2 while black is x = y line. “m” is the slope if the regression is forced through origin.

A GWR model was then developed using residual PM_2.5_ (LME estimated PM_2.5_–observed PM_2.5_) and AOD as shown in “Model development” section. The model results were then compared with ground observations to evaluate the model performances. Scatter plots between modelled and observed PM_2.5_ concentrations for the model fitting and cross-validation of stage-2 and stage-3 models are shown in Fig. 2.

Overall coefficient of determination (r^2^) values for model fitting were 0.56 and 0.60 for stage-2 and stage-3 models, respectively. The RMSE values also decreased from 22.63 to 21.63 µg m^−3^ from stage-2 to stage-3 indicating that the overall prediction accuracy increased after using the GWR model. However, CV r^2^ values were 0.51 and 0.55 for stage-2 and stage-3 models while the respective CV RMSE values were 23.91 µg m^−3^ and 22.92 µg m^−3^. The r^2^ value decreases for CV than for model fitting and the corresponding RMSE value increases for both models indicating the model slightly over fitted at both the stages. Also, after fitting the GWR model slope was increased to 0.58 from 0.55 (Fig. 2) in model fitting and to 0.54 from 0.52 (Fig. 2) in model cross validation and reduced the intercept from 23.99 to 22.97 and from 25.38 to 24.33 in model fitting and cross validation, respectively of the linear regression between model estimated and observed PM_2.5_ over MP for 2018 and 2019. Standard errors and p-values for the linear regression are provided in Supplemental Table S4. Modelled PM_2.5_ for days with surface PM_2.5_ concentration more than 100 µg m^−3^ were underestimated by both models in model fitting and cross-validation, and with increasing concentration underestimation also increased. This may be because the model was developed with most of the points below 100 µg m^−3^, therefore, less weighted is given to points with such high concentration. One more possible explanation could be that such high concentrations were local and were not reflected in a coarse resolution of 0.03° to 0.03°.

Station-wise r^2^ and RMSE for model fitting and CV for stage-2 and stage-3 models are shown as Table 3. CV r^2^ value between observed and modelled PM_2.5_ varied from 0.359 to 0.689 while RMSE varied from 15.83 to 35.85 µg m^−3^. Further, r^2^ value increased at every station after using the GWR model. In order to assess the usefulness of imputed AOD values in estimating surface PM_2.5_, we selected days when MAIAC AOD was missing but surface PM_2.5_ data were available.

**Table 3:** Summary statistics of LME and LME + GWR mode (MT is model training and CV is cross validation).

Station	N		LME			LME + GWR		
			r^2^	RMSE (µg m^−3^)	MAE (µg m^−3^)	r^2^	RMSE (µg m^−3^)	MAE (µg m^−3^)
Bhopal	106	MT	0.530	23.50	20.19	0.584	22.47	19.30
		CV	0.516	23.26	19.73	0.565	22.29	18.90
Damoh	353	MT	0.609	24.51	20.70	0.643	23.45	19.81
		CV	0.605	27.52	23.83	0.637	26.38	22.85
Dewas	729	MT	0.385	18.57	13.21	0.424	17.75	12.63
		CV	0.387	19.04	13.73	0.420	18.24	13.15
Indore	106	MT	0.450	24.67	19.95	0.498	23.55	19.05
		CV	0.469	25.59	20.85	0.512	24.51	19.97
Jabalpur	104	MT	0.644	26.02	22.68	0.690	24.88	21.69
		CV	0.644	31.12	26.71	0.687	29.83	25.60
Maihar	323	MT	0.343	21.05	16.68	0.364	20.14	15.96
		CV	0.340	22.02	17.55	0.359	21.10	16.82
Mandideep	726	MT	0.481	20.93	14.84	0.524	20.00	14.18
		CV	0.470	21.28	15.15	0.511	20.38	14.51
Pithampur	728	MT	0.524	16.08	11.78	0.558	15.37	11.25
		CV	0.514	16.53	12.27	0.545	15.83	11.76
Ratlam	316	MT	0.402	18.63	14.35	0.435	17.81	13.73
		CV	0.406	18.41	14.27	0.438	17.65	13.68
Singrauli	711	MT	0.652	34.32	25.99	0.701	32.80	24.84
		CV	0.633	37.41	27.80	0.689	35.85	26.64
Ujjain	720	MT	0.573	19.27	12.93	0.614	18.42	12.36
		CV	0.571	19.35	12.987	0.611	18.541	12.44

**Figure 3:**
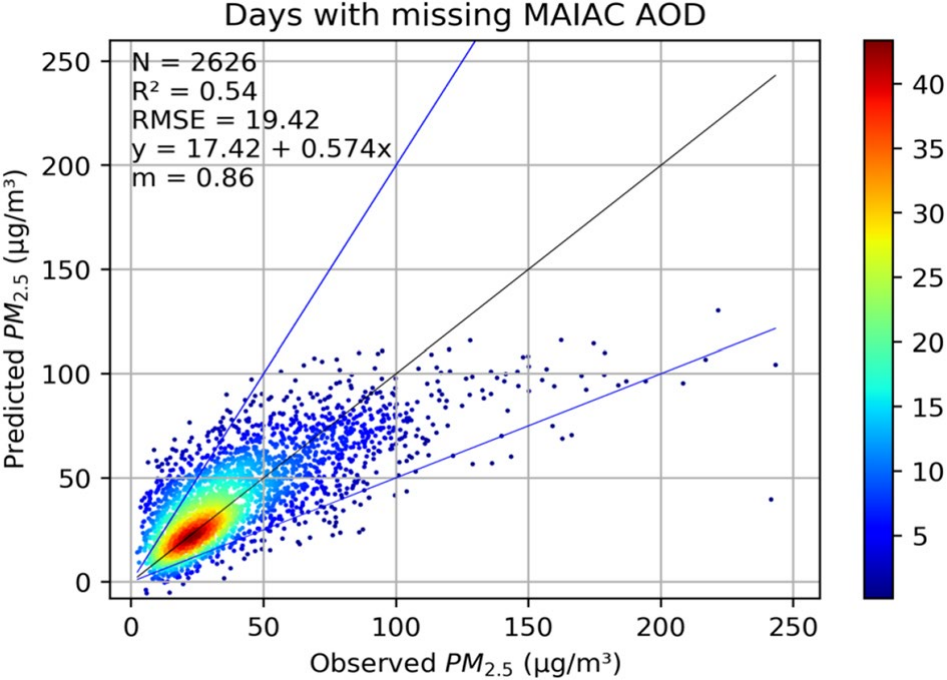
Comparison between the final model predicted PM_2.5_ using tenfold cross-validation and surface concentration on days where MAIAC AOD was unavailable. c-axis shows the point count and the blue lines are y = 2 × and y = x/2 while the black is x = y line. “m” is the slope if the regression is forced through origin.

Modelled PM_2.5_ data on only those days were selected and compared with observed values using statistical metrics (r^2^, RMSE, MAE) and a scatter plot is provided in Fig. 3. The agreement between modelled and observed PM_2.5_ on such days was good (r^2^ = 0.54) and the overall model RMSE value was 19.42 µg m^−3^ clearly indicating the usefulness of imputed AOD to predict surface PM_2.5_. We also fitted stage-2 and stage-3 models using 0.03° × 0.03° MERRA-2 AOD instead of imputed AOD and the results are discussed in Supplemental Text S2.

### Spatial distribution of surface PM_2.5_

**Figure 4:**
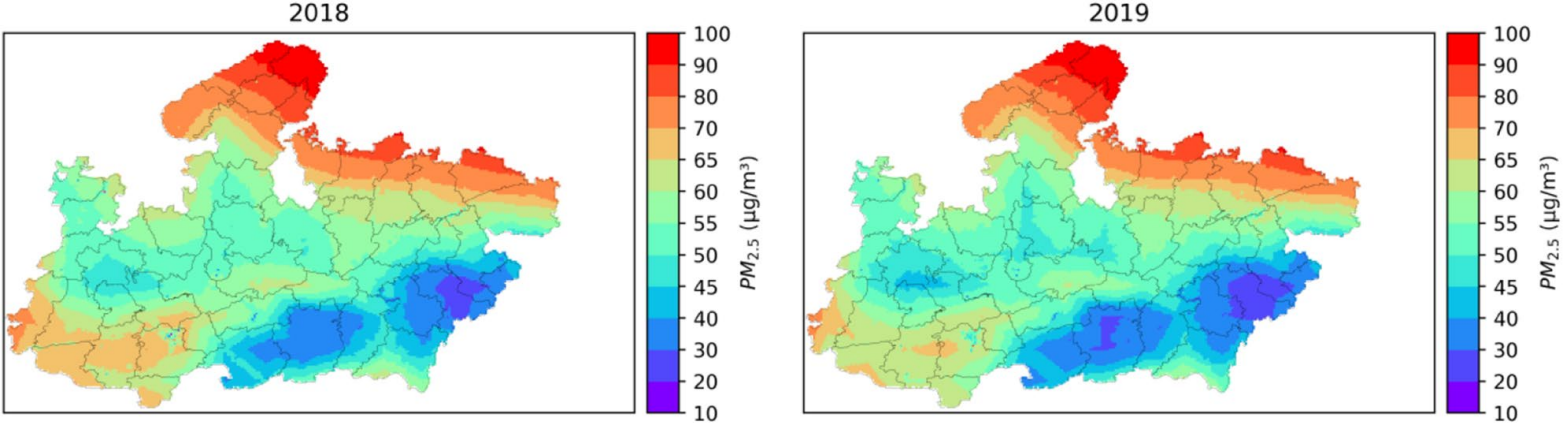
Spatial distribution of annual mean of daily surface PM_2.5_ over Madhya Pradesh for 2018 and 2019. The figure was generated using Python (version 3.7, https://www.python.org/).

Daily surface PM_2.5_ maps at a spatial resolution of (0.03° × 0.03°) were generated using the stage-3 model over Madhya Pradesh for 2018 and 2019. This data was then used to estimate the annual average of daily surface PM_2.5_ concentration and the final maps are presented in Fig. 4.

The annual average daily PM_2.5_ concentration over MP varied from 22.73 to 95.24 µg m^−3^ with a mean value of 58.19 µg m^−3^ during 2018 and from 20.80 to 96.72 µg m^−3^ with a mean value of 56.32 µg m^−3^ during 2019. It was observed that the topography of MP had a strong influence on the surface PM_2.5_, with the highest concentration in northeast MP which is a part of the Indo-Gangetic Plain (IGP) and high PM_2.5_ mass loading downstream of the Narmada valley, while locations at a high elevation (Dindori, Mandla, Amarkantak) had the least PM_2.5_ mass loading. It is also worth noting that the IGP is highly industrialized and very high population density region potentially leading to high PM_2.5_ mass loading while districts of Dindori and Mandla are the least industrially developed with huge forested cover (Kanha National Park) with negligible anthropogenic activities, potentially leading to a cleaner environment when compared with the rest of MP.

#### Seasonal PM_2.5_ maps

**Figure 5:**
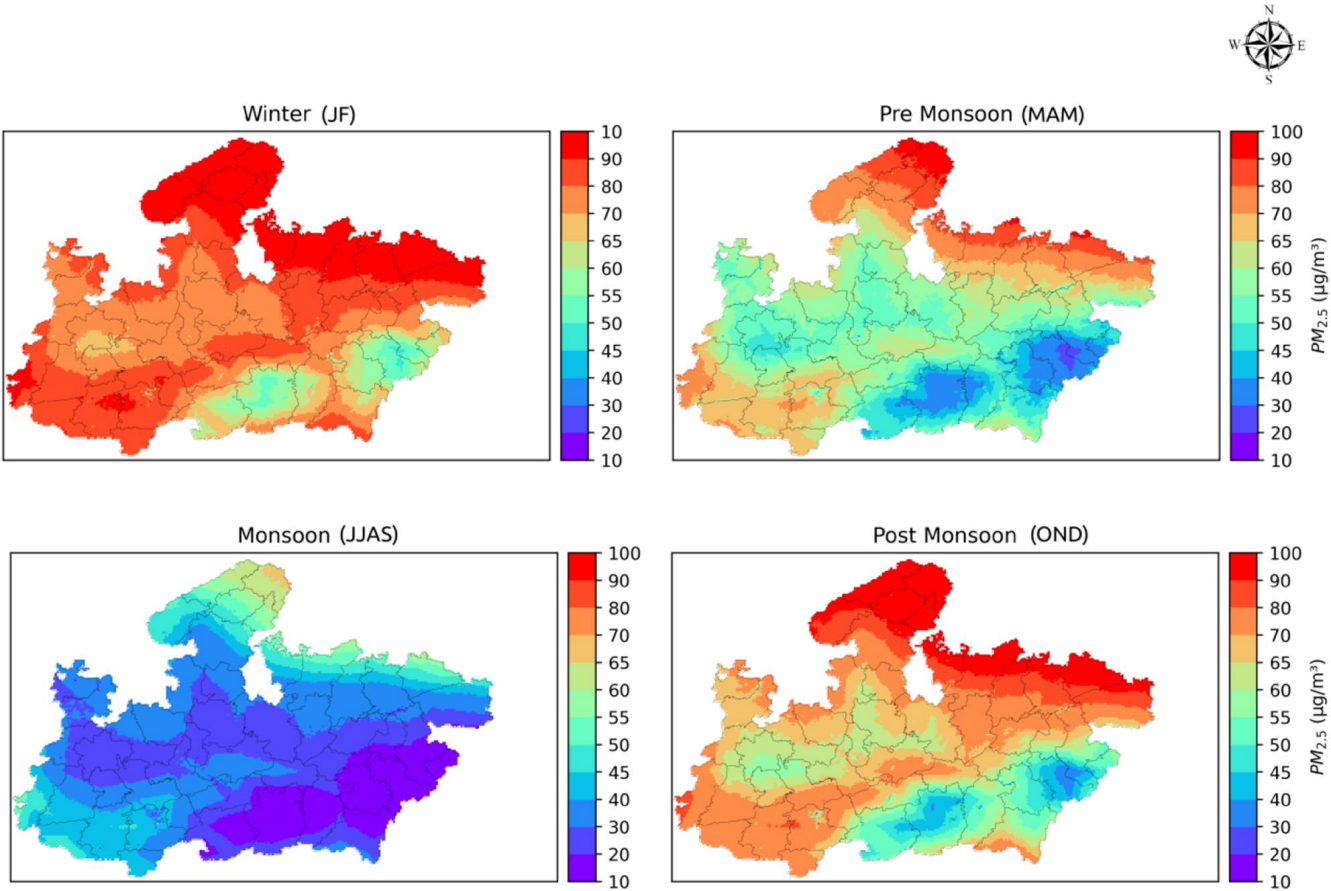
Season average (2018 and 2019) map of daily PM_2.5_ concentration over Madhya Pradesh. The figure was generated using Python (version 3.7, https://www.python.org).

To examine the seasonal variation of surface PM_2.5_ over MP, mean seasonal maps were generated utilizing the estimated daily PM_2.5_ maps for both 2018 and 2019, for a given season (Fig. 5).

PM_2.5_ mass was highest in the winter season (2018 and 2019 taken together) throughout MP with a mean value of 82.54 µg m^−3^ and the lowest concentration was estimated during the monsoon season with a mean value of 32.10 µg m^−3^. Mean PM_2.5_ concentrations in pre-monsoon and post-monsoon season were 60.14 µg m^−3^ and 71.51 µg m^−3^, respectively. Very high PM_2.5_ concentrations in northeastern MP and in Narmada valley during the post-monsoon and winter season can be attributed to crop residue burning during these seasons and stable atmosphere in low-lying areas. Low PM_2.5_ concentrations throughout MP during the monsoon season are arguably due to wet deposition of atmospheric aerosols and change in synoptic meteorology fetching a lower load of anthropogenic aerosols than during other seasons.

### Population exposure to surface PM_2.5_

Integrated Exposure–response function (IER)^40^ has been used widely to estimate the age and cause-specific mortality associated with exposure to PM_2.5_ concentrations. IER estimates the risk function for a particular disease as a function of PM_2.5_ concentrations based upon previous health studies. Equations (4)–(5) shows the IER framework that accounts for the dependence of relative risk (RR) on PM_2.5_ concentrations, Cn.

4 $$For \, Cn < Cn_{cf} ,\;\;RR_{i} \left( {Cn} \right) \, = \, 1$$

5 $$For \, Cn > Cn_{cf} , \;\;RR_{i} \left( {Cn} \right) = 1 + \alpha _{i} \left[ { \, 1 \, - \, \exp \left\{ { - \gamma_{i} \left( {Cn \, - \, Cn_{cf} } \right)^{\delta i} } \right\}} \right]$$

where. RR is the relative risk of ith disease for exposure to PM_2.5_ concentration of Cn, Cn_cf_, counterfactual concentration below which there is no associated risk due to PM_2.5_ (RR = 1) and ɑ_i_, γ_i_ and δ_i_ are disease-specific parameters. Previous studies have reported that Lung Cancer (LNC), Ischemic Heart Disease (IHD), Chronic Obstructive Pulmonary Disease (COPD) and Stroke deaths account for 97% of total deaths due to air pollution^41,42^. Therefore in this study, we have estimated district-wise premature mortality in the adult population (age > 25 years) due to LNC, IHD, COPD and Strokes using RR values provided in the look-up table by Apte et al.^43^. In that study, age-independent RR values for LNC and COPD, and age-dependent RR values for IHD and stroke for various PM_2.5_ concentrations were generated using the mean of 1000 IER curves^44^ and Cn_cf_ was taken as 5.8 µg m^−3^. Disease-specific premature mortality for the ith district and jth age group was then calculated using Eq. (6).

6 $$\Delta M \, = \, BM_{j,k} \times \, \left( {RR_{i,k} - \, 1/RR_{i,k} } \right) \, \times \, Pop_{i,j}$$

where BM_j,k_ is the disease-specific baseline mortality rate for the jth age group for kth year obtained from GBD India Compare Data Visualization (ICMR, PHFI, and IHME; 2019) for the year 2018 and 2019, RR_i,k_ is relative risk for the kth year over ith district and Pop_i,j_ is the total population of the ith district in the age group “j”. Disease-wise baseline mortality rate was provided with 95% CI which was then translated to a 95% confidence interval for disease-specific mortality over the MP. Further details on the data source and method are given in the Supplemental Text S3. Finally, the total premature mortality was calculated by adding premature mortality due to individual disease over MP.

**Figure 6:**
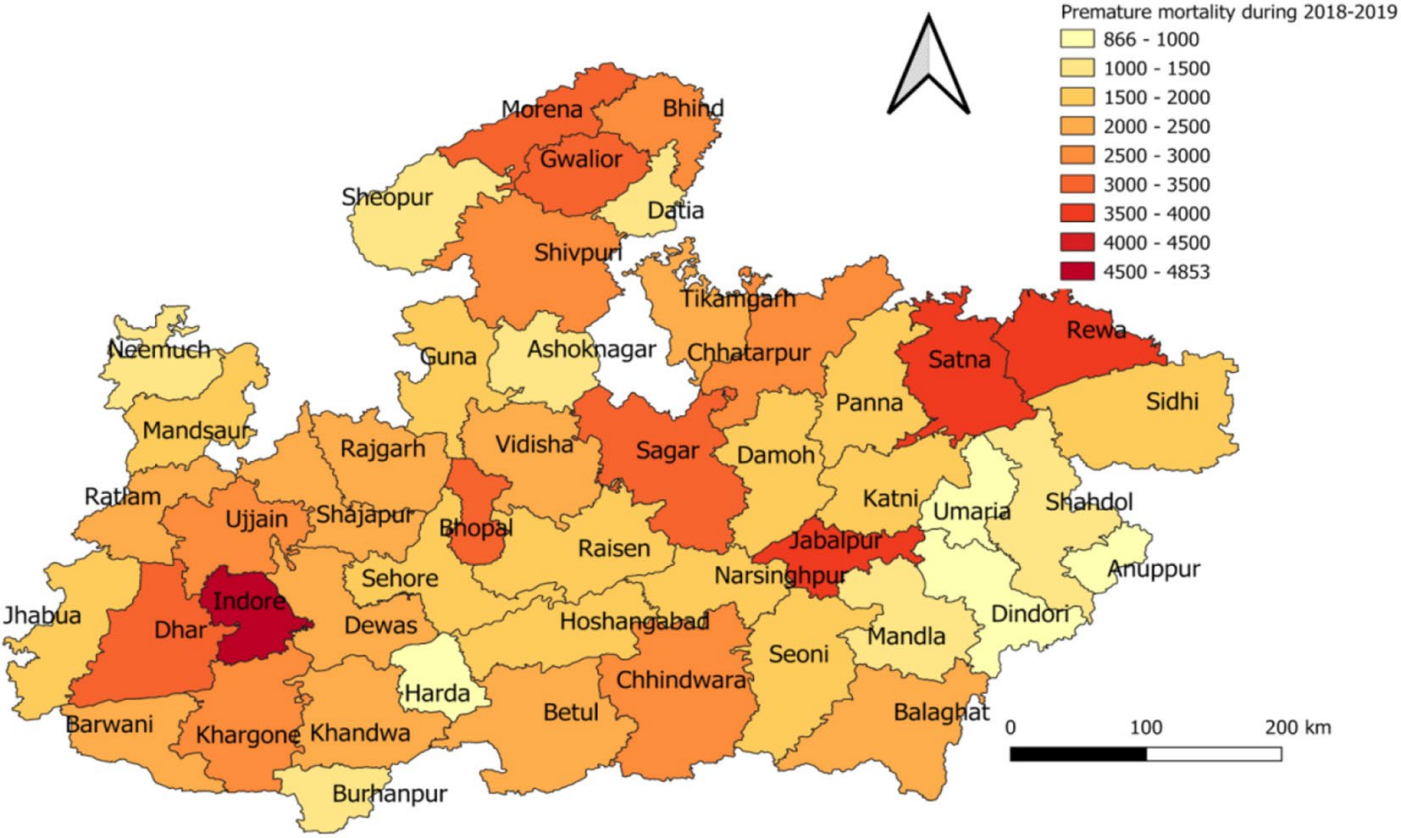
Total premature deaths in MP due to exposure to ambient PM_2.5_ concentration during 2018–2019. This map is generated using QGIS 2.18.1 (http://www.qgis.org).

District-wise population-weighted PM_2.5_ concentrations are shown in Supplemental Figure S17. The total combined (2018 and 2019) premature mortality due to exposure to PM_2.5_ concentrations in MP is estimated to be 106,115.2 (85,717.46, 127,604.6) at 95% CI including deaths due to COPD 11,720.5 (8777.197, 14,114.21), IHD 55,501.79 (45,256.85, 66,811.22), LNC 1245.11 (1010.62, 1498.76) and strokes 37,467.7 (30,672.47, 45,180.44). IHD is the major cause of premature mortality in MP causing 52.3% of total deaths followed by Strokes (35.4%), COPD (11.04) and LNC (1.17%). Indore city, which is the commercial capital of MP, with very high population density, tops the number of premature deaths due to air pollution with 4853.30 (3921.40, 5836.10) total deaths are 2018–2019 followed by Rewa, Jabalpur, Satna and Sagar. Disease-wise cause specific death for every city is provided in Supplemental Table S8. Cause-specific deaths for the top 5 cities in MP are shown in Supplemental Figure S18. Total premature mortality during 2018–2019 for districts in MP is shown in Fig. 6.

## Comparison with a CTM-satellite AOD based global PM_2.5_ estimate

**Figure 7:**
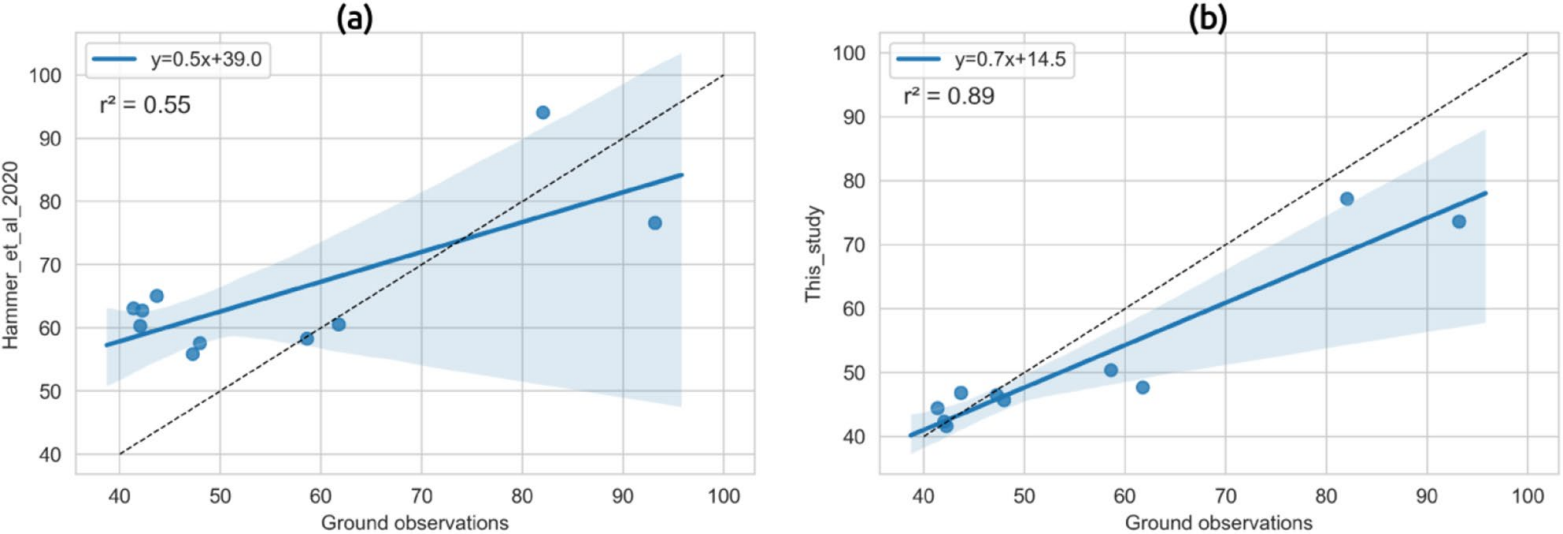
Scatter plots between model derived annual mean concentration of daily surface PM_2.5_ and ground observations (**a**) Hammer et al.^27^ model and (**b**) the model used in this study. The black dashed line shows x = y and the translucent band represents 95% CI.

An ancillary goal of this study was to assess the usefulness of our model in estimating surface PM_2.5_ compared to other CTM output-satellite AOD approaches. In order to do so, we benchmarked annual surface PM_2.5_ estimated in this study for locations with surface measurements in MP (Fig. 1) against recent estimates over the same locations derived from Hammer et al.^27^. Our model results agreed better with surface measurements (r^2^ = 0.89) compared to the CTM output-satellite AOD estimates (r^2^ = 0.55) (See Fig. 7). Also, to understand the spatial variability in surface PM_2.5_ over MP estimated by the two approaches (this study and Hammer et al.^27^), difference maps for 2018 and 2019 were generated (Supplemental Figure S19). These maps suggest that the CTM based approach did not satisfactorily capture the spatial variation in surface PM_2.5_ over MP, while over-predicting PM_2.5_ over elevated regions and under-predicting its concentrations over the Narmada valley.

## Conclusions

This study developed a three-stage statistical model to generate full coverage daily 0.03° × 0.03° surface PM_2.5_ maps over Madhya Pradesh for 2018 and 2019 using MAIAC AOD, meteorological parameters and land use information. On cross-validation, our final model was able to predict the surface PM_2.5_ with r^2^ of 0.55 and RMSE of 22.92 µg m^−3^. Mean daily averaged PM_2.5_ concentration decreased from 58.19 µg m^−3^ during 2018 to 56.32 µg m^−3^ during 2019, over MP. Winter seasons had the highest PM_2.5_ loading with a mean concentration of 82.54 µg m^−3^ (average of winter 2018 and 2019) and as expected the lowest loading was during the monsoon seasons with a mean concentration of 32.10 µg m^−3^. Also, topography and land use has a strong influence on the surface PM_2.5_ concentration in MP. IGP and low elevation areas were the most polluted while the high elevation areas had the low PM_2.5_ concentrations. Indore city had the highest premature mortality in MP during 2018–2019 followed by Rewa, Jabalpur, Satna and Sagar illustrating the fact that air pollution and associated health burden is not only a crowded commercial city problem in MP. This observation reiterates the need for current and future air quality management strategies to focus on regional air quality issues and identify air quality management districts for meaningful and effective public health protection.

Missing AOD data is a major problem in accurately estimating the surface PM_2.5_ concentration for exposure and epidemiological studies. This study has demonstrated one approach to address that problem. Although MP is used as an illustrative example to elucidate the usefulness of the model developed in this study, the method is robust and applicable across locations in the world. However, during the course of conducting this study, it was observed that the rationale for choice of locations for the CPCB stations is neither clearly documented nor obvious. It appears that the choice of location is driven by considerations of determining NAAQS violations, logistics and ease of operation. For instance, the data used in training the LME model in this study was from stations that cannot be classified as either urban hotspots or regional background locations. Thus, a country-wide network of ground monitoring stations, carefully situated in accordance with network design rules with sufficient density to capture regional and/or urban aerosols are essential to effectively exploit satellite products to provide reliable spatially continuous surface PM_2.5_ estimates. These estimates can then be used for planning both air quality management strategies and to enhance population exposure studies and other epidemiological models that assess the PM_2.5_ induced burden of disease. It is hoped that the availability of high time resolution surface PM_2.5_ measurements at several locations across India in conjunction with models, such as those developed in this study, will help enhance GBD exposure assessment estimates for this region in the future.

## Supplementary Information

Supplementary Information.

## Data Availability

There are no linked research data sets for this submission. All data used in this study are publicly available. Web links/citations as appropriate to the data used are listed in the manuscript.
