# Supplementary material for: Population exposure across central India to PM2.5 derived using remotely sensed products in a three-stage statistical model
Source: Sci Rep. 2021 Jan 12;11:544. doi: 10.1038/s41598-020-79229-7 (PMC7804491; doi:10.1038/s41598-020-79229-7)
Supplement: Supplementary file 1 — Supplementary Information. [file 41598_2020_79229_MOESM1_ESM.pdf]

Supplemental Information

Population exposure across central India to $PM_{2.5}$ derived using remotely sensed products in a three-stage statistical model

PREM MAHESHWARKAR[1] AND RAMYA SUNDER RAMAN[1, 2*]

[1]Department of Earth and Environmental Sciences
[2]Center for Research on Environment and Sustainable Technologies

Indian Institute of Science Education and Research Bhopal
Bhopal Bypass Road, Bhauri, Bhopal – 462 066, Madhya Pradesh, INDIA

[*] Corresponding author. Phone: 91-755-269 1371; Fax: 91-755-269 2392
Email: ramyasr@iiserb.ac.in

Supplemental Text

All tables and figures referred to in the supplemental text are only supplemental tables and figures, as the case may be. Further, all supplemental figures are tables are labeled and organized in the chronological order of their appearance in the main manuscript.

Supplemental Text S1: Imputing Missing MAIAC AOD with MERRA-2 AOD

Grid-wise linear regression was fitted between resampled MAIAC and MERRA-2 AOD for each year as discussed in Section 3.3.1 in the main manuscript. We fitted both yearly and seasonal linear regression between daily values of MAIAC and MERRA-2 AOD and are shown in supplemental Figure S7 – Figure S12. While fitting seasonal linear regression the p-values for slope were not statistically significant ($p > 0.01$) for large number of grid points in monsoon Therefore, in the final model yearly regression was fit to impute missing MAIAC AOD values.

Supplemental Text S2: LME and LME + GWR model using MERRA-2 AOD

In this section, we discuss the development of stage-2 (LME) and subsequently stage-3 (GWR) model using output from stage-2 model same as in the main manuscript but using MERRA-2 AOD instead of imputed AOD. In stage-2 the model performed very similar (see Table S5) to that of imputed AOD indicating MERRA-2 AOD was able to capture the temporal variation in MAIAC AOD (high temporal r^2 between MERRA-2 AOD and MAIAC AOD shown in Figure S5 also suggests the same) therefore performed as good as imputed AOD in stage-2 model. Scatter plot between modelled and observed $PM_{2.5}$ after stage-2 for both model fitting and CV with MERRA-2 AOD are shown in Figure S14 and the detailed statistics of regression between modelled and observed $PM_{2.5}$ is provided in Table S6

However, in stage-3 (geographically weighted regression) the slope value between $PM_{2.5}$ residual and MERRA-2 AOD was not statistically significant, indicating MERRA-2 couldn't capture spatial variability in surface $PM_{2.5}$ for ground stations in MP potentially because with coarse spatial resolution and the AOD generated from CTM is spatially smoothed (and hence not able to capture the grid cell variability that is evident in MAIAC AOD) see Figure S15. Average AOD over the study period from both MERRA-2 and MAIAC are shown in Figure S15 and scatter plot between grid values of remapped MAIAC and MERRA-2 is shown in Figure S16

The grid cell-wise correlation coefficient of 2018 and 2019 means of MAIAC and MERRA-2 AOD was 0.56. However, while MERRA-2 AOD values agree with large MAIAC AOD values, the agreement breaks down at small (< 0.4) values (see Figure S16). Therefore, in the final model, imputed AOD was used to estimate surface $PM_{2.5}$ concentrations over MP for 2018-2019.

Supplemental Text S3: Population Exposure to Surface $PM_{2.5}$

To estimate the population exposure to surface $PM_{2.5}$ and associated premature mortality in MP following datasets were used -

Population

1. Estimated district-wise population for 2020 was obtained from http://www.populationu.com/in/madhya-pradesh-population. The year 2020 was the nearest available dataset to the study period, therefore, the total district-wise population for 2020 was used for both 2018 and 2019
2. Demographic data for India was obtained from United Nations, Department of Economic and Social Affairs, World Population Prospect, 2019 https://population.un.org/wpp/Download/Files/1_Indicators%20(Standard)/EXCEL_FILES/1_Population/WPP2019_POP_F15_1_ANNUAL_POPULATION_BY_AGE_BOTH_SEXES.xlsx. Demographic data over India was then used to estimate the district-wise age group specific population over MP. Here, we have assumed that demography of each district is the same as that of India.

Baseline mortality

1. Baseline mortality data over MP for LNC, IHD, COPD and stokes for 2018 and 2019 were obtained from GBD India Compare Data Visualisation (ICMR, PHFI, and IHME; 2019) https://vizhub.healthdata.org/gbd-compare/india and is provided in Table S7.

Relative risk

1. The relative risk (RR) for each disease was obtained from (Apte et al. 2015). Apte et al. 2015 have provided age-independent relative risk for Chronic Obstructive Pulmonary Disease (COPD) and Lung cancer (LNC) and age-dependent relative risk values for Ischemic Heart Disease (IHD) and Strokes. These RR values are provided from 1 μgm^{-3} to 410 μgm^{-3} at an interval of 0.1 μgm^{-3}.

Assumptions:

1 The cause-specific baseline death rate was available at the state level therefore, we have assumed that the baseline mortality rate does not change with districts

2. Baseline mortality data for Strokes and IHD were available at a coarser resolution (age < 5, 15-49, 50-70 and 70+ years) whereas this study was conducted for adults at every 5-year gap (25-30, 30-35, 35-40, 40-45, 45-50, 50-55, 55-60, 60-65, 65-70, 70-75, 75-80, and 80+) therefore, the baseline mortality rate for overlapping groups was assumed to be same.

Supplemental Tables

Table S1.Summary of national studies to estimate surface $PM_{2.5}$ concentrations in India.

Region	Study period	No of ground monitors	Method	r^2	$PM_{2.5}/PM_{10}$	References
India	March 2000-February 2010	6	CTM	0.49 (Kanpur, Delhi)	Monthly	Dey et al., 2012
India	2016	41	MLR	0.40	Daily	Sathe et al., 2019
India	2011	15	CTM	0.35	Monthly	Krishna et al., 2019
India	Jan 2017-Aug 2017	33	STMEM	0.75	Daily	Unnithan et al., 2020
India	2015	21	GAM	0.71	Daily	Sahu et al., 2020

Table S2. Station-wise and season-wise AOD and $PM_{2.5}$ over stations (average value from 2018-2019) mean (standard deviation)

Station	Winter (JF)		Pre-Monsoon (MAM)		Monsoon (JJAS)		Pot-Monsoon (OND)	
	MAIAC AOD	$PM_{2.5}$	MAIAC AOD	$PM_{2.5}$	MAIAC AOD	$PM_{2.5}$	MAIAC AOD	$PM_{2.5}$
Bhopal	0.374 (0.188)	83.79 (23.23)	0.370 (0.178)	- (-)	0.567 (0.375)	14.96 (5.89)	0.440 (0.178)	70.58 (28.75)
Damoh	0.403 (0.256)	77.59 (29.36)	0.357 (0.128)	41.22 (10.97)	0.528 (0.402)	18.31 (13.02)	0.402 (0.221)	61.94 (23.32)
Dewas	0.398 (0.203)	59.94 (19.65)	0.362 (0.132)	45.7 (13.91)	0.456 (0.188)	27.98 (17.90)	0.391 (0.155)	56.45 (20.51)
Indore	0.389 (0.203)	73.13 (22.35)	0.376 (0.119)	-	0.400 (0.123)	19.63 (7.92)	0.410 (0.156)	61.07 (22.55)
Jabalpur	0.441 (0.274)	105.97 (26.88)	0.363 (0.125)	-	0.469 (0.209)	14.58 (5.46)	0.461 (0.206)	71.55 (28.45)
Katni	0.422 (0.252)	123.11 (36.12)	0.375 (0.140)	-	0.46 (0.231)	17.06 (7.35)	0.469 (0.219)	92.26 (38.64)
Maihar	0.406 (0.265)	37.72 (17.33)	0.398 ;(0.160)	30.09 (9.07)	0.640 (0.549)	18.77 (9.35)	0.507 (0.340)	29.20 (9.92)
Mandideep	0.388 (0.192)	71.82 (36.54)	0.374 (0.186)	56.81 (40.14)	0.464 (0.260)	25.51 (14.41)	0.437 (0.166)	66.06 (33.58)
Pithampur	0.395 (0.217)	66.16 (33.24)	0.373 (0.101)	41.57 (10.16)	0.394 (0.119)	24.35 (10.41)	0.428 (0.172)	58.04 (20.25)
Ratlam	0.333 (0.165)	61.14 (19.18)	0.334 (0.09)	45.18 (10.74)	0.340 (0.150)	28.69 (15.41)	0.390 (0.204)	58.66 (16.22)
Singrauli	0.398 (0.252)	138.53 (58.62)	0.408 (0.160)	83.41 (23.02)	0.523 (0.276)	43.72 (74.82)	0.586 (0.356)	106.93 (41.99)
Ujjain	0.394 (0.221)	78.56 (37.08)	0.330 (0.104)	54.02 (41.96)	0.488 (0.188)	23.64 (9.86)	0.435 (0.185)	61.36 (31.12)

Table S3. Detailed statistics from stage-2 model

	Coefficient	Std.Err.z	Z	P>|z|	[0.025	0.975]
Intercept	-823.031	79.194	-10.393	0.000	-978.248	-667.814
AOD	34.833	2.159	16.133	0.000	30.601	39.065
V10	-1.178	0.186	-6.341	0.000	-1.542	-0.814
RH100	-23.502	0.988	-23.791	0.000	-25.438	-21.566
Temperature	-1.104	0.098	-11.280	0.000	-1.295	-0.912
Pressure	0.009	0.001	11.730	0.000	0.008	0.011
Urban	0.100	0.005	21.603	0.000	0.091	0.109
Grassland	0.796	0.051	15.490	0.000	0.695	0.897

Table S4. Fitting coefficient between predicted and estimated $PM_{2.5}$ using LME and LME+GWR model

Model	Variable	Coefficient	Std error	p-Value	0.025	0.975
LME_MT	Intercept	23.994	0.439	<0.001	23.135	24.856
	Slope	0.556	0.007	<0.001	0.542	0.570
LME_CV	Intercept	23.382	0.457	<0.001	24.486	26.278
	Slope	0.522	0.007	<0.001	0.508	0.537
GWR_MT	Intercept	22.97	0.420	<0.001	22.148	23.794
	Slope	0.575	0.007	<0.001	0.563	0.589
GWR_CV	Intercept	24.339	0.438	<0.001	23.480	25.118
	Slope	0.542	0.007	<0.001	0.528	0.556

Table S5. Comparison between stage-2 model performance of imputed and MERRA-2 AOD

Statistical metrics	Stage-2 model training (Imputed AOD)	Stage-2 model training (MERRA-2 AOD)	Stage-2 cross validation (Imputed AOD)	Stage-2 cross validation (MERRA-2 AOD)
r^2	0.56	0.54	0.51	0.50
RMSE	22.63	22.82	23.91	24.16
slope and intercept	0.015 +0.988x	0.015 +0.986x	0.015 +0.988x	0.015 +0.986x

Table S6. Fitting coefficients between predicted and estimated $PM_{2.5}$ using MERRA-2 AOD for LME model

Model	Variable	Coefficient	Std error	p-Value	0.05	0.095
LME_MT MERRA-2 AOD	Intercept	24.075	0.445	<0.001	23.204	24.947
	Slope	0.555	0.007	<0.001	0.541	0.569
LME_CV MERRA-2 AOD	Intercept	25.52	0.461	<0.001	24.623	26.430
	Slope	0.520	0.007	<0.001	0.506	0.535

Table S7. Cause specific baseline mortalityrate with 95% confidence interval per 100000 deaths over Madhya Pradesh

Year	Age (years)	IHD	Strokes	COPD	LNC
2018	<5	0	0.97(0.63, 1.45)	65.71(49.75, 78.34)	5.46(4.84, 6.51)
2018	5-14	0	0.28(0.19, 0.4)	65.71(49.75, 78.34)	5.46(4.84, 6.51)
2018	15-49	23.46(19.17, 28.69)	7.32 (5.87, 8.98)	65.71(49.75, 78.34)	5.46(4.84, 6.51)
2018	50-69	281.77(232.18, 339.15)	164.12(135.35, 197.03)	65.71(49.75, 78.34)	5.46(4.84, 6.51)

2018	70<	1137.17(938.87, 1357.89)	712.73(585.18, 837.83)	65.71(49.75, 78.34)	5.46(4.84, 6.51)
2019	<5	0	0.92(0.6, 1.41)	66.41(49.18 80.78),	5.63(4.52, 6.84)
2019	5-14	0	0.28(0.18, 0.4)	66.41(49.18 80.78),	5.63(4.52, 6.84)
2019	15-49	23.27(18.74, 28.71)	7.23(5.73, 8.83)	66.41(49.18 80.78),	5.63(4.52, 6.84)
2019	50-69	279.44(223.43, 339.88)	161.64(129.67, 198.29)	66.41(49.18 80.78),	5.63(4.52, 6.84)
2019	70<	113.67(934.41, 1338.6)	711.07(581.64, 838.04)	66.41(49.18 80.78),	5.63(4.52, 6.84)

Table S8. Cause specific mortality, population and population weighted concentration of surface PM2.5 for every district in MP during 2018-2019

DISTRICT	PWC	Population	COPD			IHD			LNC			Strokes			TOTAL		
Anuppur	35.45042	854130	89.55786	67.07509	107.8378	518.3067	422.6239	624.0135	9.422633	7.648634	11.34058	333.9972	272.1222	400.8528	951.2843	769.4699	1144.045
Ashoknagar	54.97565	963381	135.7172	101.6306	163.4421	658.1419	536.6444	792.2596	14.41992	11.70319	17.35747	448.099	365.0709	537.7703	1256.378	1015.049	1510.829
Balaghat	50.7466	1939936	259.6273	194.444	312.6292	1299.739	1059.833	1564.639	27.55249	22.36443	33.16166	880.4589	717.3485	1056.634	2467.378	1993.99	2967.063
Barwani	65.56329	1579904	247.6275	185.4505	298.1892	1122.132	915.0222	1350.737	26.35643	21.39275	31.72319	767.7933	625.553	921.3918	2163.91	1747.419	2602.041
Betul	41.2726	1795913	209.6488	157.0306	252.4227	1139.578	929.2471	1371.922	22.14922	17.98061	26.65581	754.6262	614.8537	905.6255	2126.002	1719.112	2556.626
Bhind	93.9088	1941426	372.8507	279.1785	449.058	1475.247	1202.939	1775.633	39.68852	32.20801	47.77766	1002.782	816.9877	1203.366	2890.568	2331.313	3475.834
Bhopal	51.67761	2703010	365.8893	274.0259	440.5863	1818.818	1483.101	2189.501	38.83381	31.52135	46.73985	1233.66	1005.116	1480.505	3457.202	2793.765	4157.333
Burhanpur	63.32373	863946	132.6335	99.33067	159.715	609.078	496.6601	733.1688	14.11162	11.45402	16.98502	416.5724	339.3986	499.911	1172.396	946.8434	1409.78
Chhatarpur	74.96737	2009108	340.5524	255.004	410.1444	1466.144	1195.502	1764.771	36.26713	29.43254	43.65759	1002.732	816.9412	1203.338	2845.695	2296.88	3421.911
Chhindwara	36.61377	2383651	255.6181	191.468	307.7627	1460.332	1190.797	1758.142	26.91839	21.85292	32.39441	947.0138	771.6202	1136.52	2689.882	2175.739	3234.819
Damoh	57.59477	1441210	208.9207	156.4476	251.6009	995.1243	811.4205	1197.898	22.21432	18.02904	26.73981	678.9355	553.137	814.7936	1905.195	1539.034	2291.032
Datia	85.50529	896900	163.6867	122.5638	197.1422	670.6507	546.8545	807.2242	17.4319	14.14638	20.98473	457.3135	372.581	548.7954	1309.083	1056.146	1574.146
Dewas	52.73061	1782635	244.5259	183.1371	294.4406	1205.622	983.0975	1451.324	25.96324	21.07477	31.24843	818.8432	667.1525	982.6775	2294.954	1854.462	2759.69
Dhar	59.29244	2491804	367.4677	275.1999	442.4988	1731.451	1411.866	2084.246	39.08059	31.72062	47.03822	1182.395	963.3422	1418.958	3320.394	2682.129	3992.741
Dindori	35.88769	803157	84.89611	63.58632	102.2206	489.0277	398.7566	588.7609	8.933682	7.252053	10.7517	315.853	257.3452	379.0697	898.7105	726.9402	1080.803
Khandwa	64.04547	1493470	230.9097	172.9293	278.0594	1055.572	860.7437	1270.625	24.56893	19.94179	29.57183	722.0646	588.2945	866.5191	2033.115	1641.909	2444.775
Guna	56.04022	1415332	201.7263	151.069	242.9239	971.1221	791.8616	1169.013	21.43913	17.40091	25.8054	661.7854	539.174	794.2049	1856.073	1499.505	2231.948
Gwalior	82.48455	2316521	414.5313	310.3933	499.2495	1721.1	1403.401	2071.608	44.14379	35.82415	53.14023	1174.787	957.1192	1409.798	3354.562	2706.737	4033.796
Harda	59.10478	650330	95.76893	71.72187	115.324	451.6353	368.2732	543.6595	10.18313	8.265312	12.25668	308.3899	251.2566	370.0906	865.9772	699.5171	1041.331
Hoshangabad	52.28691	1415139	193.0998	144.6173	232.5233	955.1478	778.8451	1149.807	20.49888	16.63874	24.67241	648.3904	528.2699	778.1281	1817.137	1468.371	2185.131
Indore	54.36809	3735435	522.1647	391.0665	628.7641	2544.455	2074.811	3062.98	55.47159	45.02623	66.76484	1731.212	1410.495	2077.594	4853.302	3921.398	5836.103
Jabalpur	49.61906	2808149	370.7103	277.6157	446.4219	1871.349	1525.89	2252.765	39.32473	31.91738	47.33386	1265.629	1031.13	1518.914	3547.013	2866.553	4265.435
Jhabua	64.96921	1168555	182.155	136.4164	219.3498	828.3729	675.4792	997.1346	19.38812	15.73666	23.33612	566.742	461.7469	680.1221	1596.658	1289.379	1919.943
Katni	53.60112	1472928	204.1574	152.8836	245.8601	1000.103	815.4787	1203.915	21.68339	17.59848	26.10028	679.9817	553.9899	816.0577	1905.925	1539.951	2291.933
Mandla	37.63367	1202592	131.6131	98.57911	158.4677	743.0637	605.9084	894.591	13.87008	11.25952	16.69231	484.5539	394.802	581.5242	1373.101	1110.549	1651.275

DISTRICT	PWC	Population	COPD			IHD			LNC			Strokes			TOTAL		
Mandsaur	54.86702	1528069	215.0045	161.0123	258.9146	1043.424	850.8144	1256.056	22.84318	18.54044	27.49545	710.3484	578.7382	852.4893	1991.62	1609.105	2394.955
Morena	88.92578	2241206	417.9402	312.9374	503.3673	1687.275	1375.819	2030.859	44.49568	36.10881	53.565	1149.149	936.2308	1379.021	3298.859	2661.096	3966.813
Narsimhapur	52.238	1244714	169.7609	127.134	204.4256	839.946	684.8999	1011.127	18.02046	14.62654	21.69	570.1745	464.5386	684.2681	1597.902	1291.199	1921.511
Neemuch	54.49802	941716	131.8998	98.77462	158.841	641.9382	523.4359	772.7558	14.01238	11.37273	16.86652	436.8685	355.9243	524.2899	1224.719	989.5076	1472.753
Panna	67.37117	1158833	184.6927	138.298	222.4333	827.9214	675.0854	996.5812	19.66268	15.95732	23.66938	566.5995	461.6144	679.9653	1598.876	1290.955	1922.649
Raisen	54.65372	1518021	212.9693	159.4933	256.4563	1035.431	844.3056	1246.436	22.62485	18.36384	27.23189	704.7166	574.1562	845.7243	1975.742	1596.319	2375.848
Rajgarh	55.3175	1762228	249.0694	186.532	299.9231	1205.314	982.837	1450.933	26.461	21.47785	31.84875	820.8286	668.7607	985.0632	2301.673	1859.607	2767.768
Ratlam	52.48171	1658779	226.7505	169.8158	273.049	1120.385	913.5765	1348.718	24.07367	19.53996	28.97553	760.7189	619.7838	912.9372	2131.928	1722.716	2563.68
Rewa	77.08727	2696221	464.3012	347.6636	559.1857	1978.016	1612.886	2380.885	49.4513	40.1318	59.52884	1352.174	1101.637	1622.685	3843.942	3102.318	4622.285
Sagar	52.65677	2711442	371.6514	278.3144	447.564	1833.152	1494.744	2206.744	39.458	32.02481	47.49521	1245.041	1014.353	1494.197	3489.303	2819.437	4196.001
Satna	68.20435	2540986	407.807	305.366	491.1395	1819.804	1483.866	2190.518	43.41514	35.23371	52.26195	1245.435	1014.669	1494.619	3516.46	2839.135	4228.538
Sehore	53.15306	1494918	206.0694	154.3331	248.137	1012.885	825.9305	1219.305	21.88258	17.76215	26.33747	688.279	560.7723	825.9923	1929.116	1558.798	2319.772
Seoni	42.9698	1572209	188.5094	141.1856	226.9865	1008.633	822.4519	1214.264	19.94017	16.186	23.99896	671.6652	547.2394	806.0794	1888.747	1527.063	2271.329
Shahdol	45.2377	1215312	150.9303	113.0314	181.7503	790.7591	644.7812	951.957	15.98231	12.97224	19.23683	530.0077	431.8095	636.0829	1487.679	1202.594	1789.027
Shajapur	53.16963	1724456	237.8784	178.1547	286.4416	1168.687	952.9734	1406.857	25.25827	20.50206	30.40056	794.1972	647.0671	953.1047	2226.021	1798.697	2676.804
Sheopur	73.21975	784162	131.1375	98.19724	157.9323	569.6162	464.4701	685.6407	13.96469	11.33327	16.8101	389.6902	317.4877	467.6516	1104.408	891.4884	1328.035
Shivpuri	66.22366	1967697	310.4357	232.4574	373.8666	1400.817	1142.226	1686.192	33.0445	26.81779	39.77755	958.5704	780.9592	1150.362	2702.868	2182.461	3250.198
Sidhi	63.6135	1284818	197.8506	148.1568	238.2709	906.7943	739.4031	1091.539	21.05203	17.08557	25.34092	620.2585	505.3347	744.362	1745.955	1409.98	2099.512
Tikamgarh	73.86774	1647489	276.9108	207.3478	333.5002	1198.803	977.507	1442.983	29.49011	23.93246	35.49981	820.052	668.1069	984.1157	2325.256	1876.894	2796.099
Ujjain	47.69379	2265025	291.2212	218.1111	350.6652	1494.031	1218.264	1798.563	30.87074	25.05846	37.15469	1006.765	820.2617	1208.218	2822.887	2281.695	3394.601
Umaria	48.69634	735024	95.80336	71.7455	115.3686	487.4441	397.4603	586.7975	10.16104	8.247157	12.23038	329.1304	268.1491	394.9985	922.5389	745.6021	1109.395
Vidisha	51.86855	1663118	225.7056	169.0265	271.8005	1120.206	913.4178	1348.508	23.96019	19.44709	28.8399	760.0607	619.2385	912.1587	2129.933	1721.13	2561.307
Khargone	64.02595	2135272	330.1405	247.2437	397.5523	1509.192	1230.639	1816.662	35.12715	28.51155	42.27999	1032.364	841.1074	1238.896	2906.824	2347.501	3495.39
TOTAL			5694.44	4264.332	6857.571	26700.47	21771.79	32140.88	605.2521	491.2336	728.5357	18163.09	14797.94	21797.28	51163.26	41325.3	61524.27

Supplemental Figures

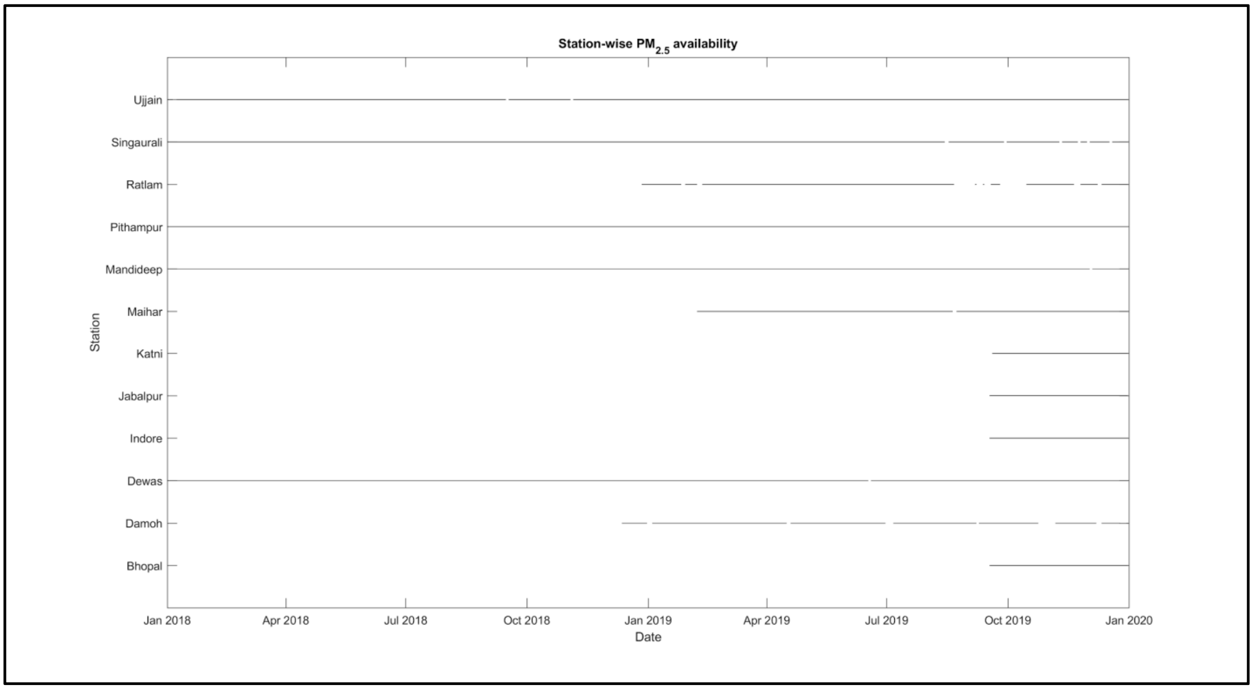

Figure S1. Station-wise availability of surface $PM_{2.5}$ data over Madhya Pradesh during the study period

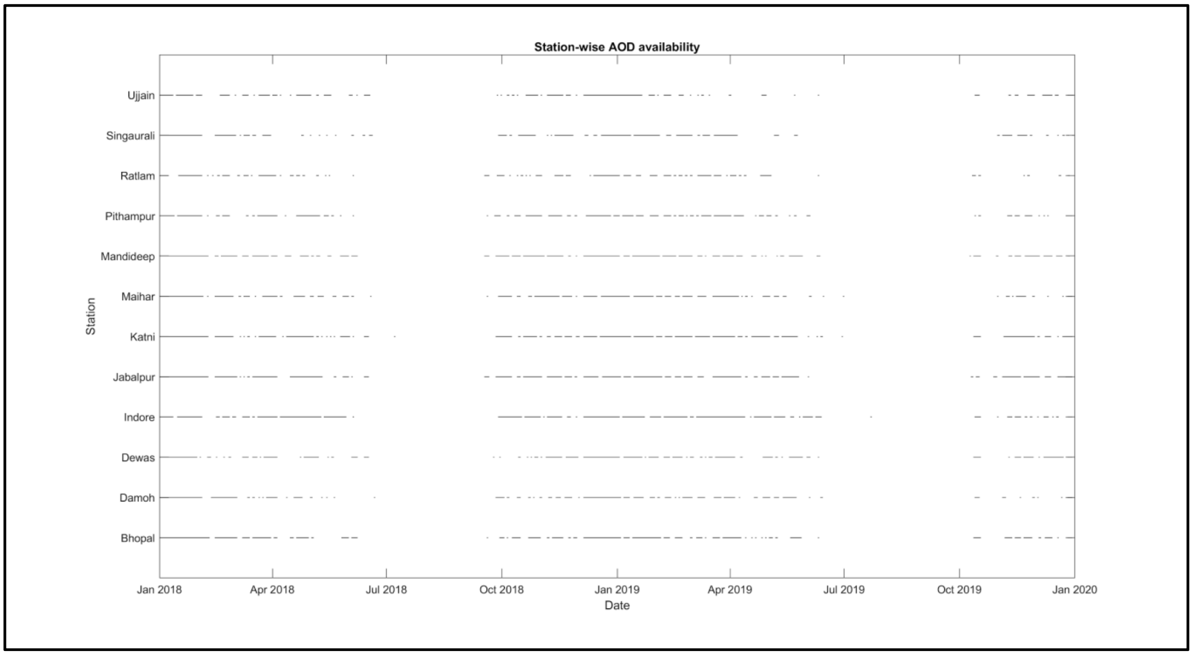

Figure S2. Availability of MAIAC AOD over surface $PM_{2.5}$ monitoring stations in Madhya Pradesh during the study period

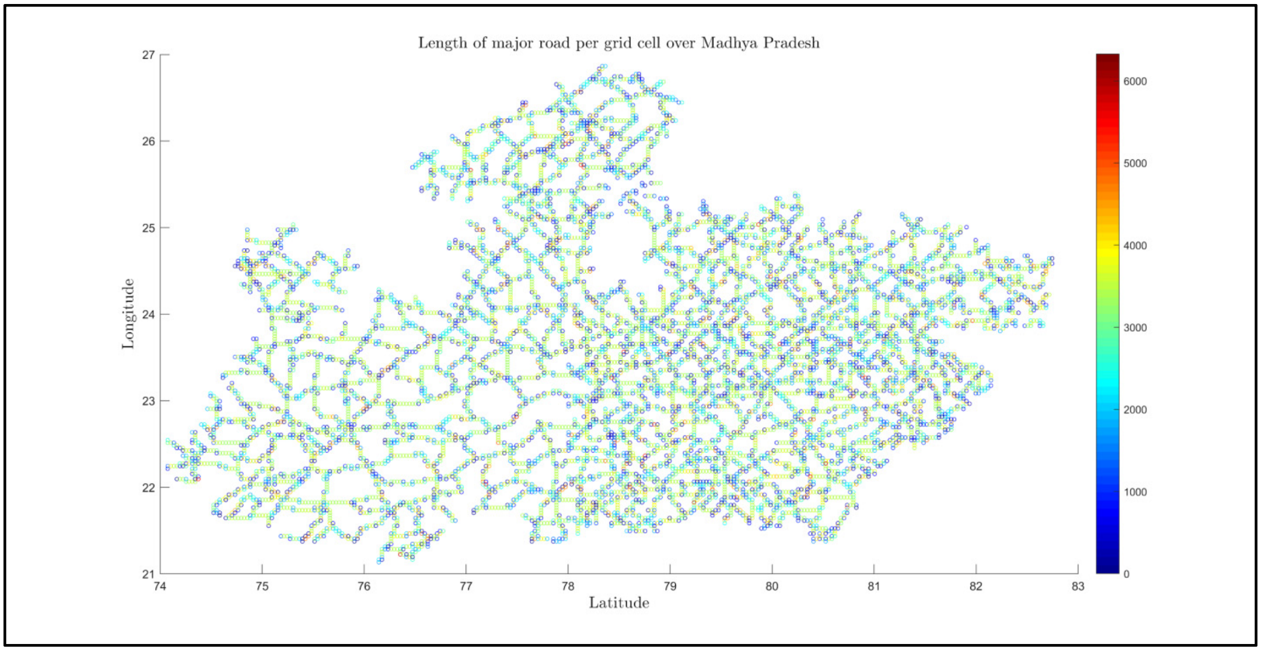

Figure S3. Major road length map of Madhya Pradesh per 0.03° x 00.3° grid cell. (scale in meters). The figure is generated using MATLAB 2017b (https://in.mathworks.com/products/matlab.html).

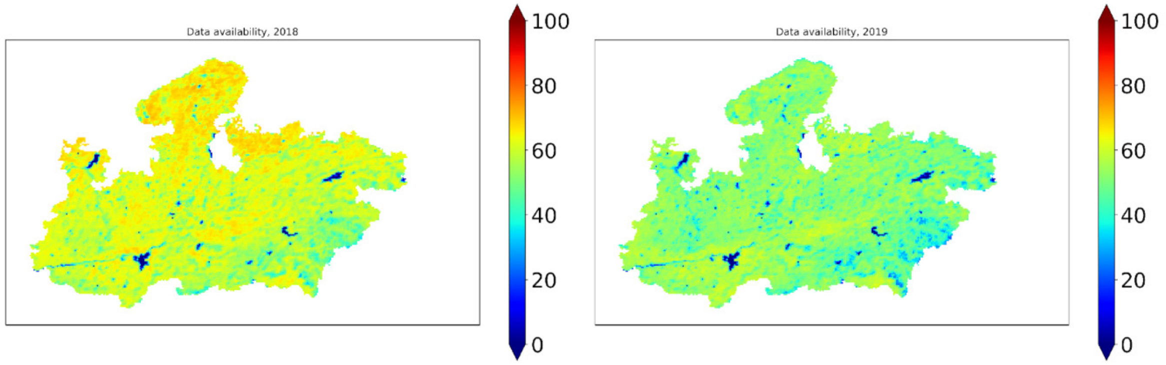

Figure S4. Percentage coverage of MAIAC AOD over the study area for 2018 and 2019. The figure was generated using Python (version 3.7, https://www.python.org/)

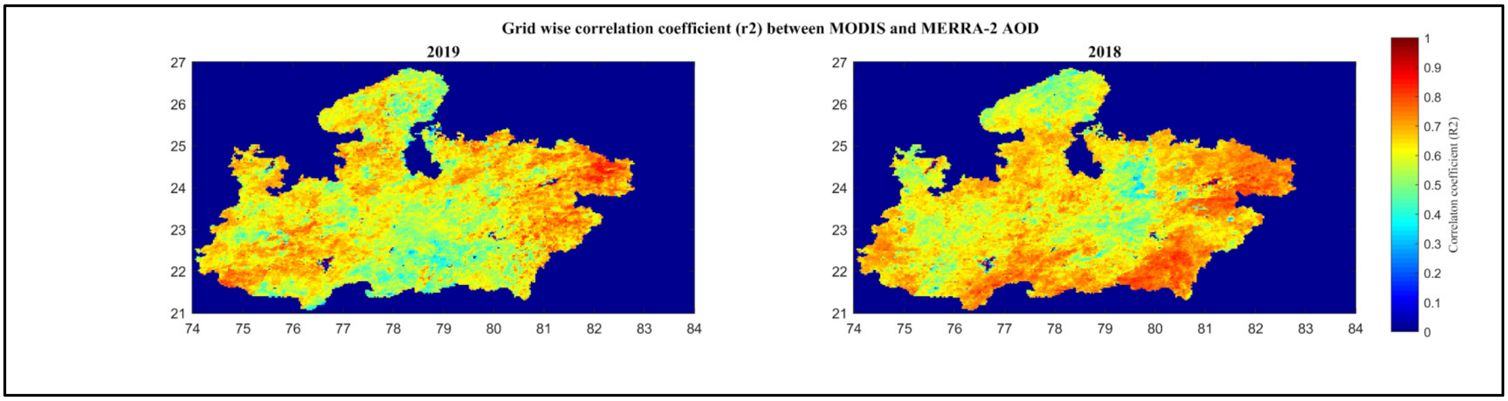

Figure S5. Yearly grid wise correlation coefficient between MAIAC and MERRA-2 AOD over the study area. The figure is generated using MATLAB 2017b (https://in.mathworks.com/products/matlab.html)

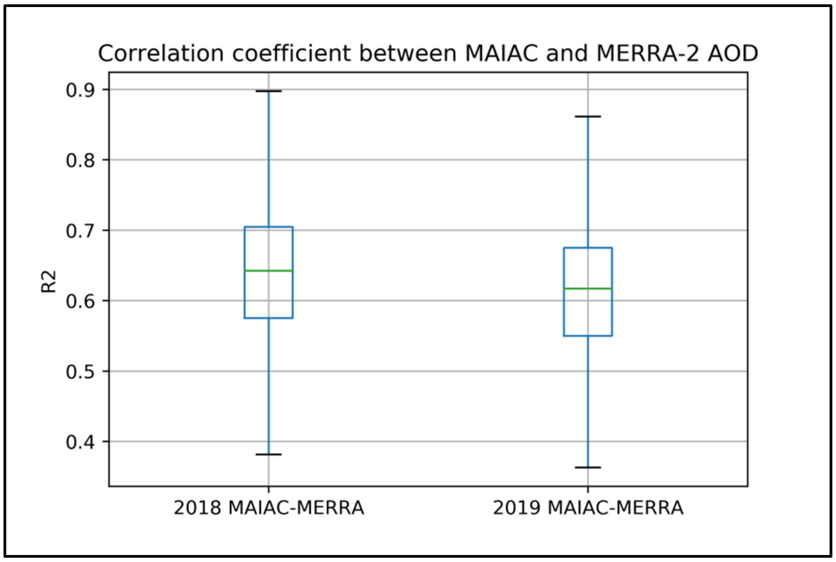

Figure S6. Boxplot of grid-wise r^2 between MAIAC and MEERA 2 AOD over the study area

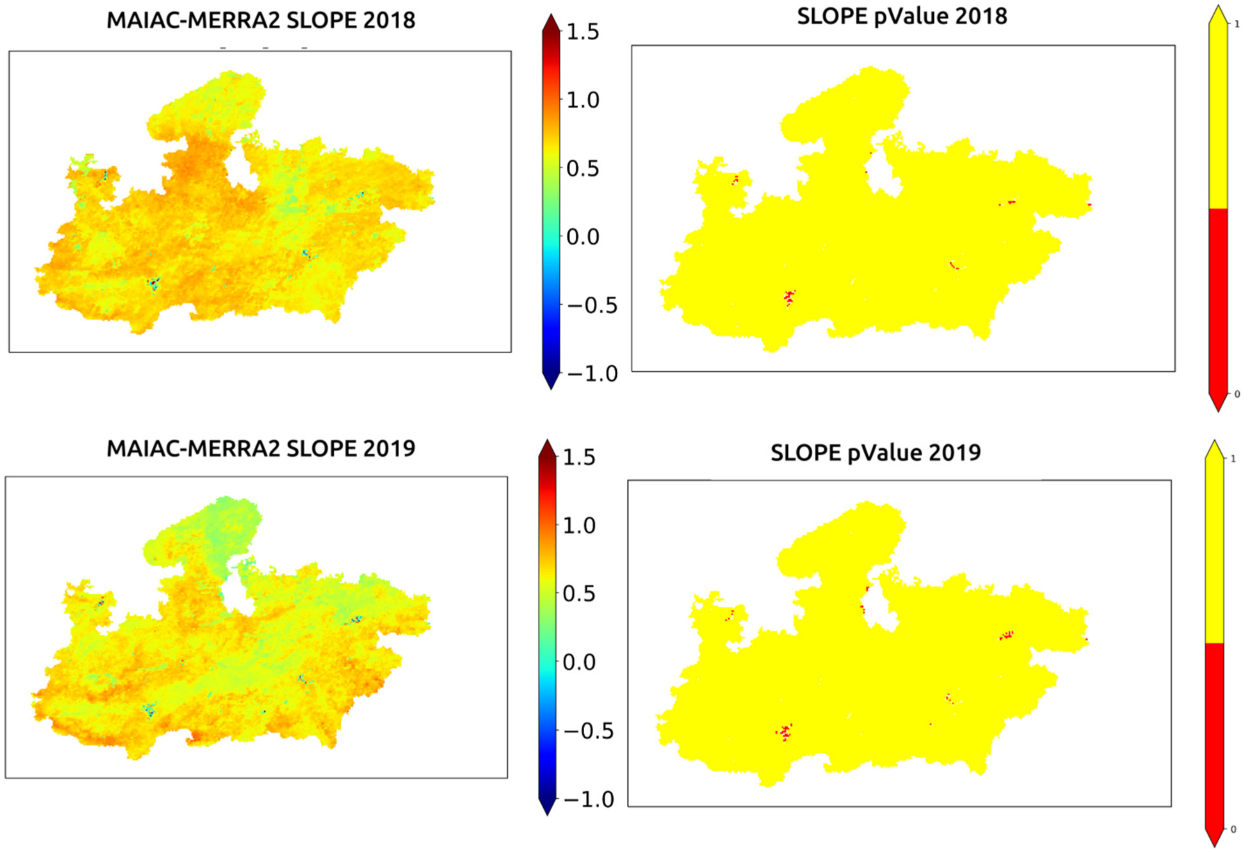

Figure S7. Grid wise slope value obtained by fitting linear regression between MERRA-2 AOD and MAIAC AOD for 2018 and 2019. Corresponding p-values are also shown in the right panel, p-values <=0.01 are plotted as 1 (yellow) while p-values > 0.01 are plotted as 0 (red). The figure was generated using Python (version 3.7, https://www.python.org/)

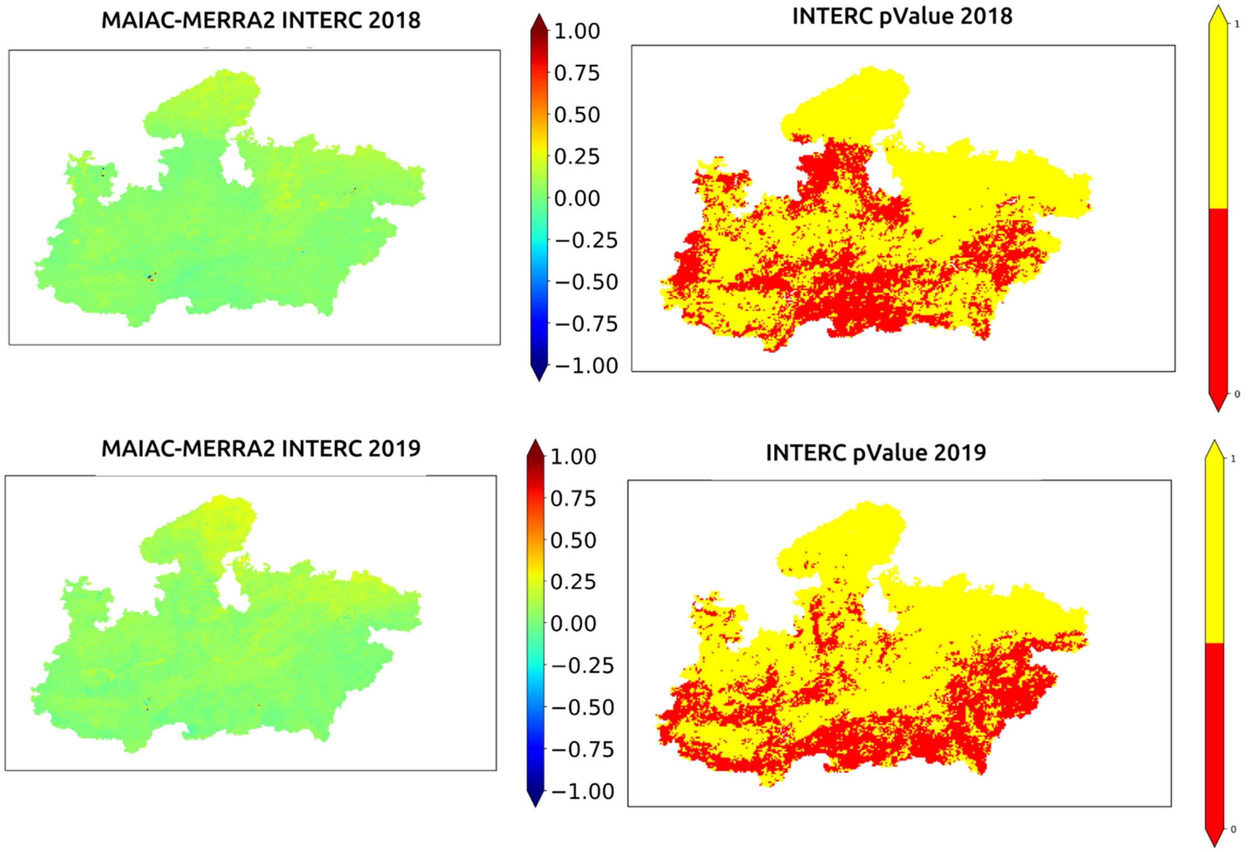

Figure S8. Grid wise intercept value obtained by fitting linear regression between MERRA-2 AOD and MAIAC AOD for 2018 and 2019. Corresponding p-values are also shown in the right panel. p-values<=0.01 are plotted as 1 (yellow) while p-values > 0.01 are plotted as 0 (red). The figure was generated using Python (version 3.7, https://www.python.org/)

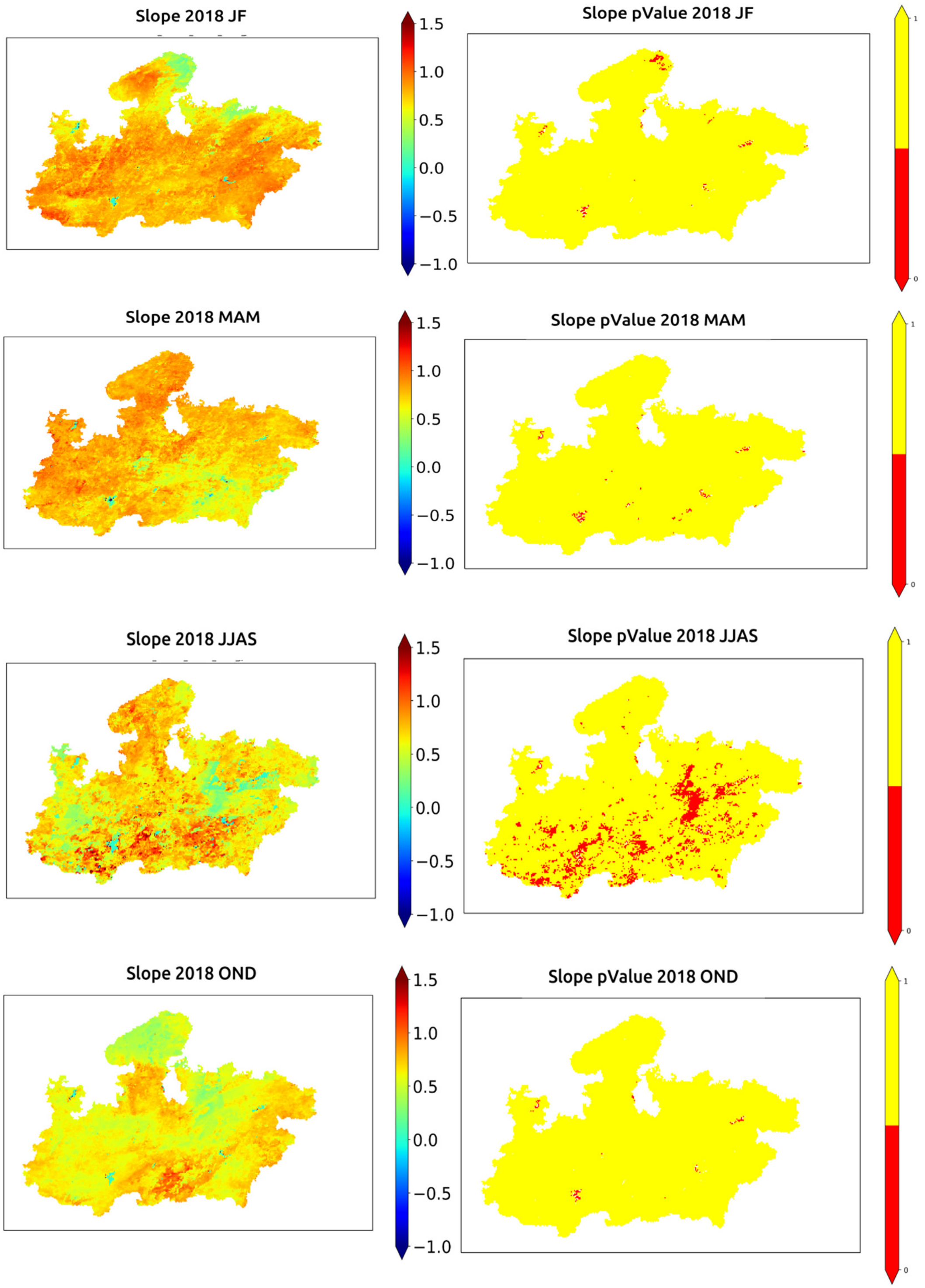

Figure S9. Grid wise slope value obtained by fitting seasonal linear regression between MERRA-2 AOD and MAIAC AOD for 2018. Corresponding p-values are also shown in the right panel. pValues<=0.01 are plotted as 1 (yellow) while p-values > 0.01 are plotted as 0 (red). The figure was generated using Python (version 3.7, https://www.python.org/)

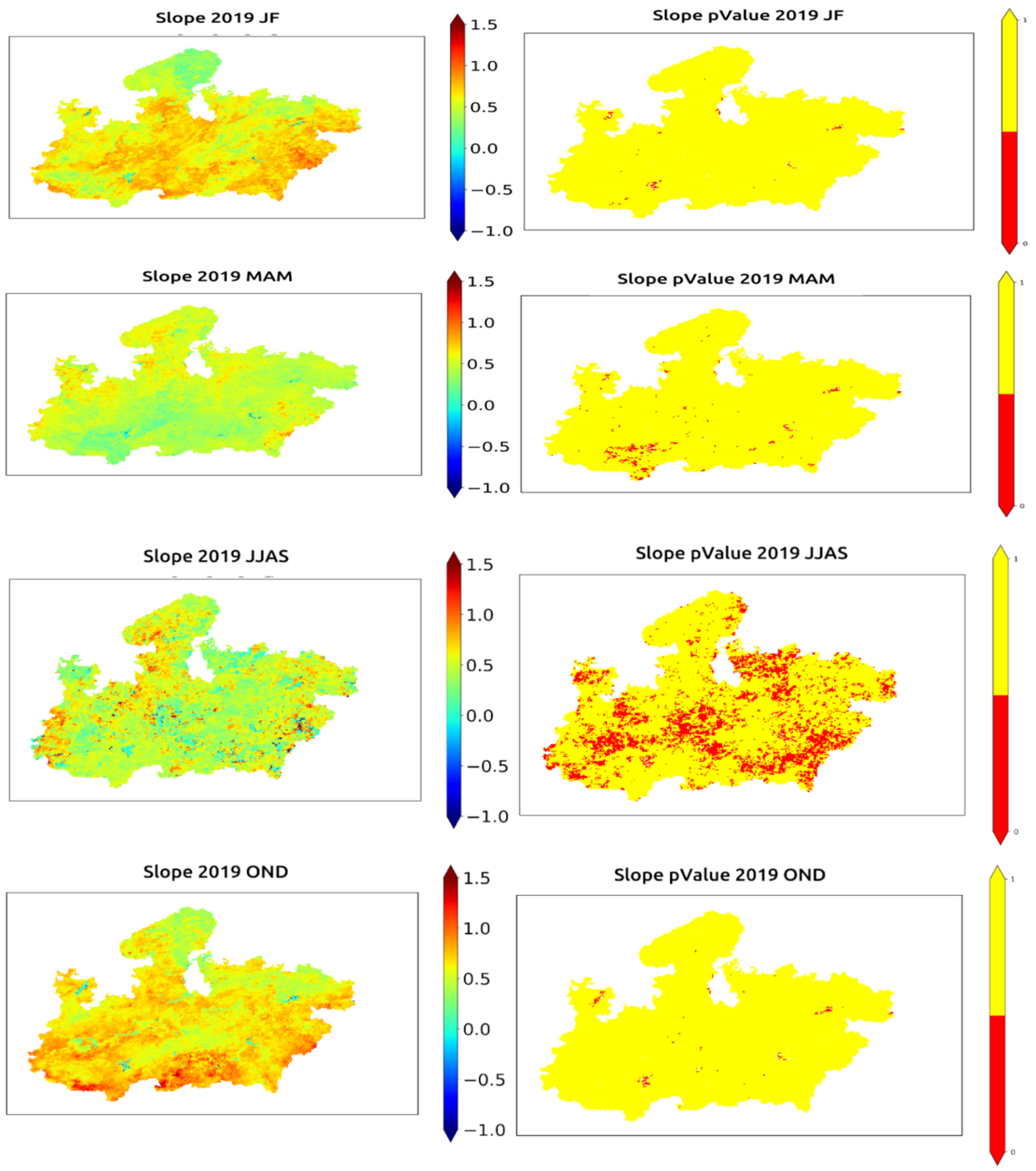

Figure S10. Grid wise slope value obtained by fitting seasonal linear regression between MERRA-2 AOD and MAIAC AOD for 2019. Corresponding p-values are also shown in the right panel. p-values<=0.01 are plotted as 1 (yellow) while p-values > 0.01 are plotted as 0 (red). The figure was generated using Python (version 3.7, https://www.python.org/)

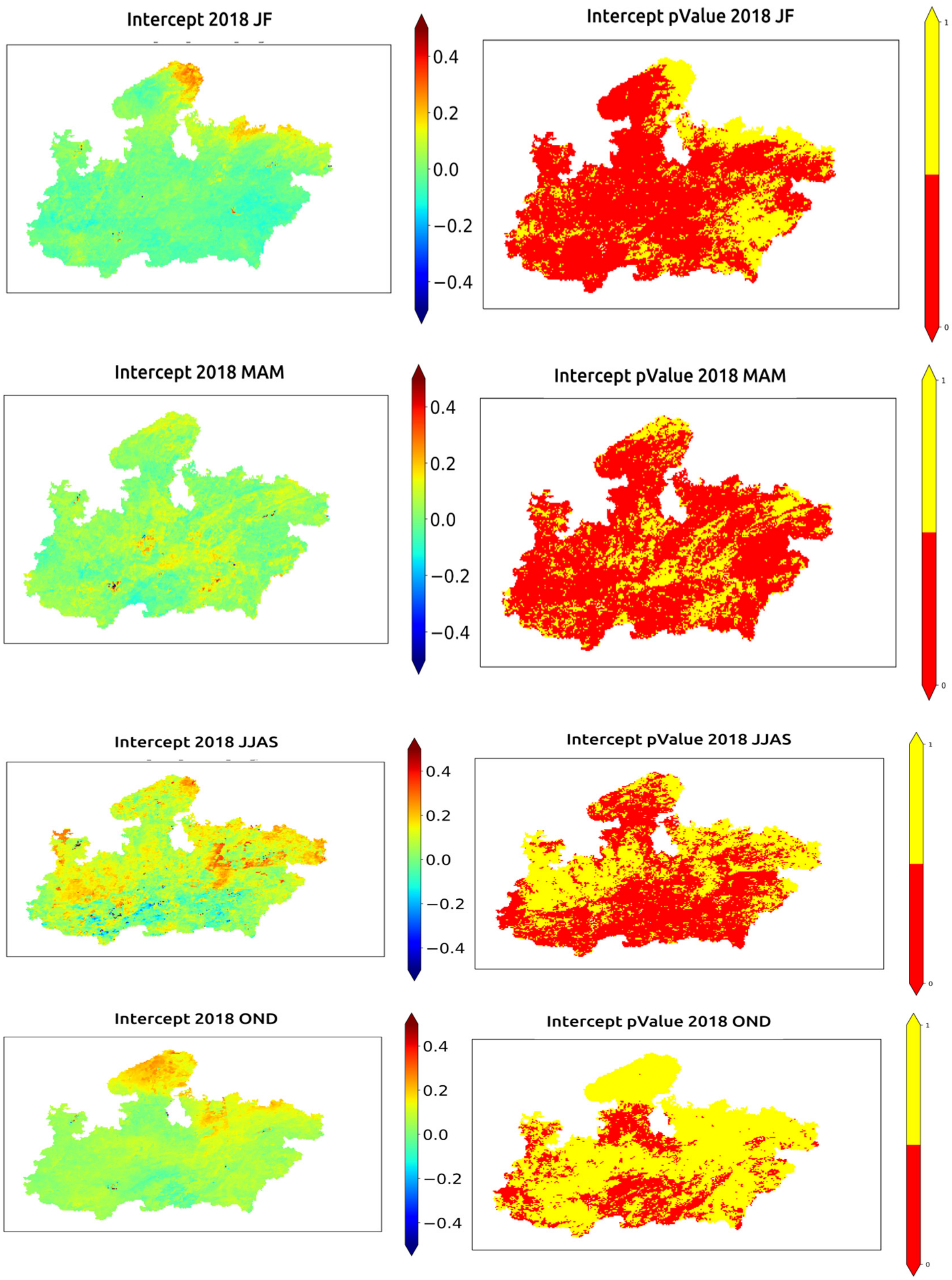

Figure S11. Grid wise intercept value obtained by fitting seasonal linear regression between MERRA-2 AOD and MAIAC AOD for 2018. Corresponding p-values are also shown in the right panel. p-values<=0.01 are plotted as 1 (yellow) while p-values > 0.01 are plotted as 0 (red). The figure was generated using Python (version 3.7, https://www.python.org/)

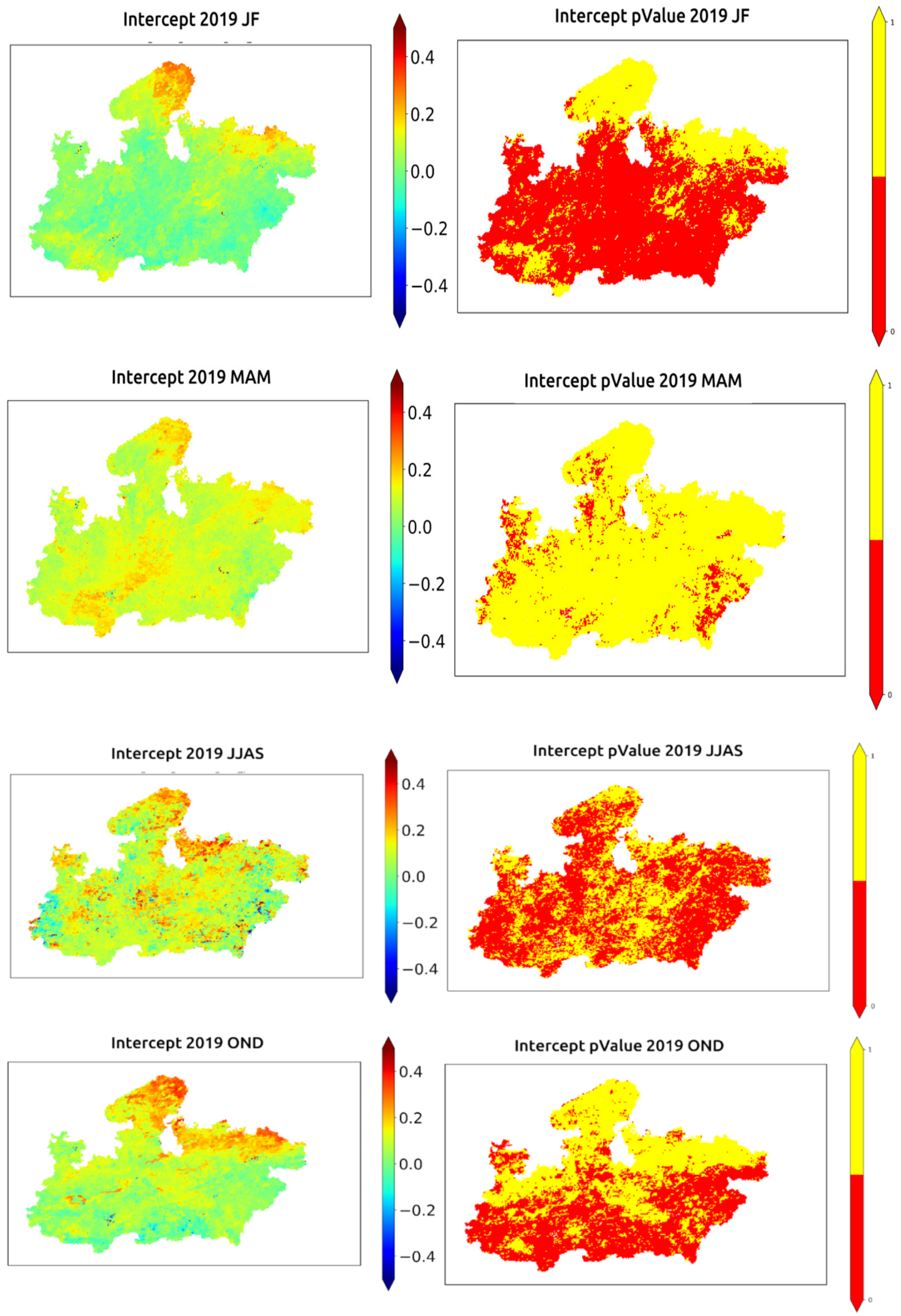

Figure S12. Grid wise intercept value obtained by fitting seasonal linear regression between MERRA-2 AOD and MAIAC AOD for 2019. Corresponding p-values are also shown in the right panel. p-values<=0.01 are plotted as 1 (yellow) while p-values > 0.01 are plotted as 0 (red). The figure was generated using Python (version 3.7, https://www.python.org/)

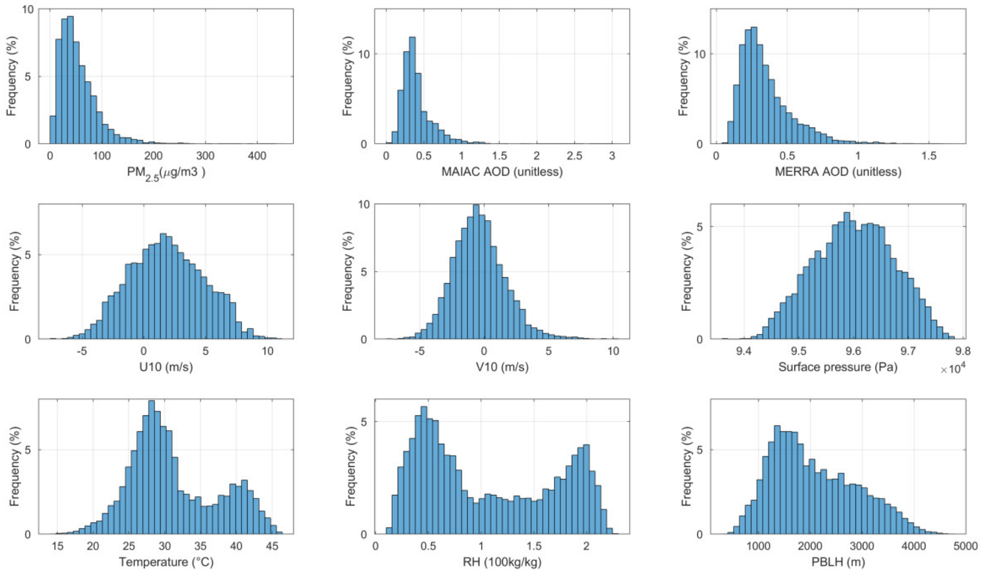

Figure S13. Distribution of AOD, PM 2.5 and meteorological variables used in the study

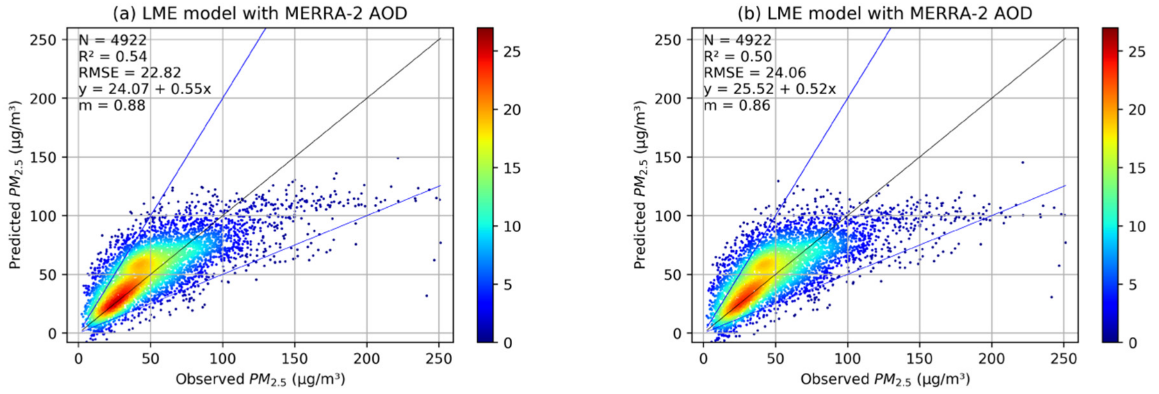

Figure S14. (a) LME and (b) LME are model training and 10 fold cross-validation of LME model using MERRA-2 AOD, respectively over Madhya Pradesh during 2018-2019. (c axis is the point count). Blue lines are y = 2x and y = x/2 while black line is y = x line. “m” is the slope if the regression is forced through origin

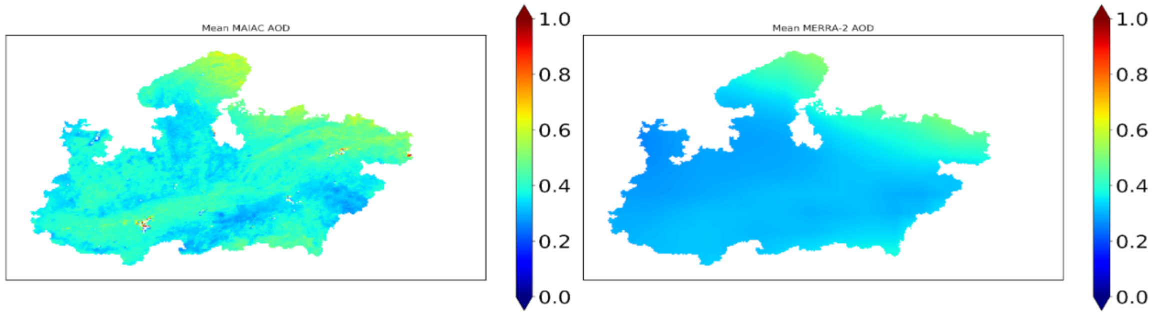

Figure S15. Average AOD over MP for 2018 and 2019. The figure was generated using Python (version 3.7, https://www.python.org/)

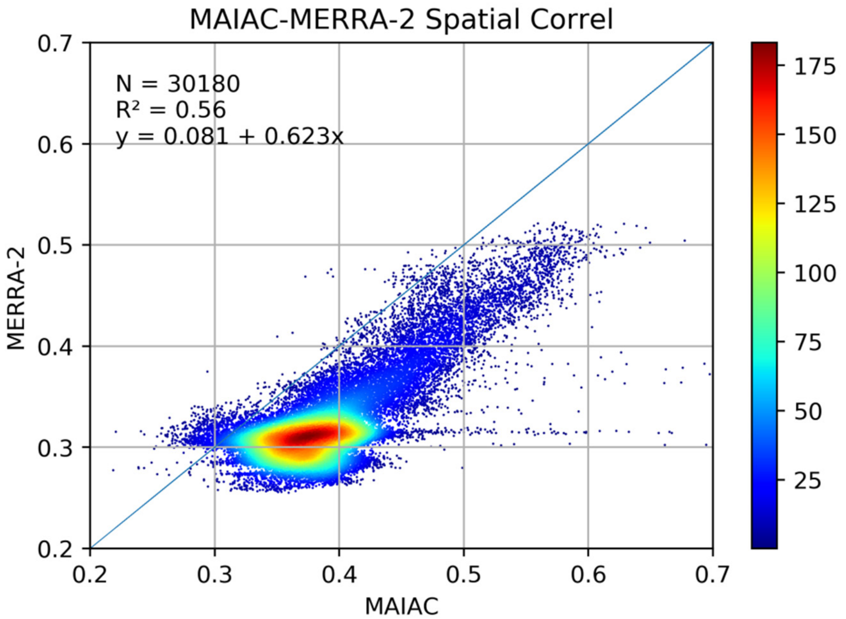

Figure S16. Scatter plot between AOD values taken average over grid cell for the study period between MERRA-2 and MAIAC AOD

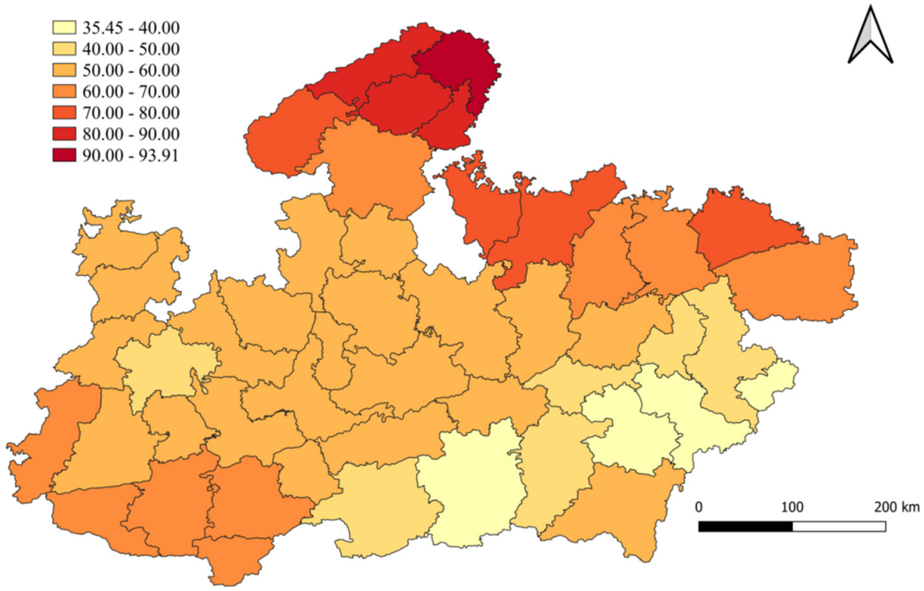

Figure S17. Population weighted $PM_{2.5}$ concentration over MP during the study period. This map is generated using QGIS 2.18.1 (http://www.qgis.org)

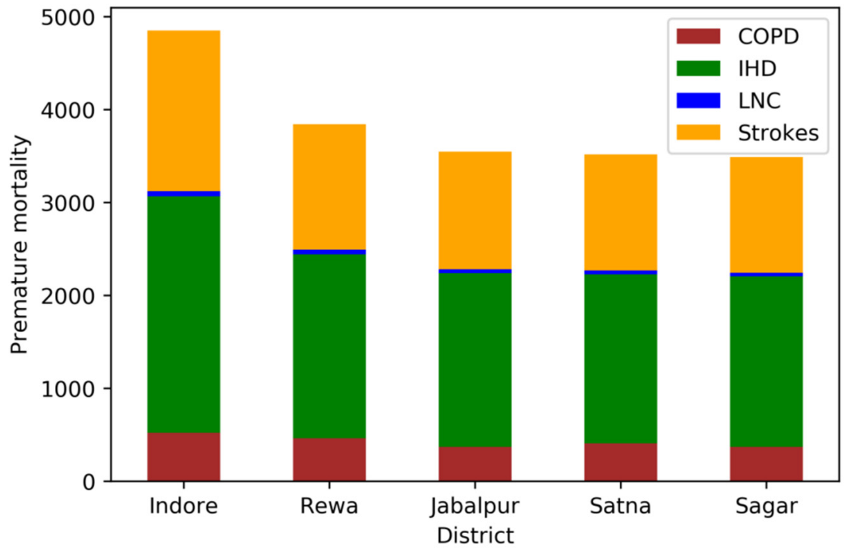

Figure S18. Cause specific premature mortality in top 5 districts ranked by total death during 2018-2019 in MP.

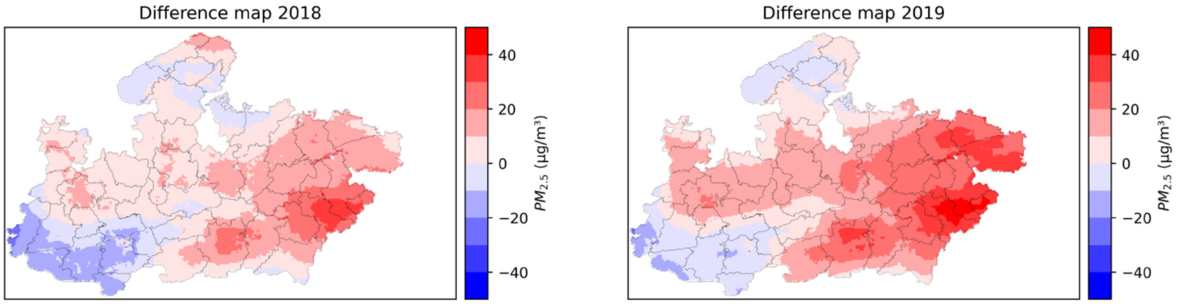

Figure S19. Difference maps between (Hammer et al., 2020 – this study) for Madhya Pradesh. The figure was generated using Python (version 3.7, https://www.python.org/)

References

Apte, J. S., Marshall, J. D., Cohen, A. J. &Brauer, M. Addressing Global Mortality fromAmbient PM2.5. *Environmental Science & Technology* vol. 49 8057–8066 (2015).

Dey, S. et al. Variability of outdoor fine particulate (PM2.5) concentration in the Indian Subcontinent: A remote sensing approach. Remote Sensing of Environment vol. 127 153–161 (2012).

Krishna, R. K. et al. Surface PM2.5 Estimate Using Satellite-Derived Aerosol Optical Depth over India. Aerosol and Air Quality Research vol. 19 25–37 (2019).

Sahu, S. K. et al. Estimating ground level PM concentrations and associated health risk in India using satellite based AOD and WRF predicted meteorological parameters. Chemosphere 255, 126969 (2020).

Sathe, Y. et al. Application of Moderate Resolution Imaging Spectroradiometer (MODIS) Aerosol Optical Depth (AOD) and Weather Research Forecasting (WRF) model meteorological data for assessment of fine particulate matter (PM2.5) over India. Atmospheric Pollution Research vol. 10 418–434 (2019)

Unnithan, S. L. K., Kesav Unnithan, S. L. & Gnanappazham, L. Spatiotemporal mixed effects modeling for the estimation of PM2.5 from MODIS AOD over the Indian subcontinent. GIScience & Remote Sensing vol. 57 159–173 (2020)
